# Genetic testing in pediatric kidney transplant recipients to promote informed choice and improve individualized monitoring

**DOI:** 10.1186/s13023-024-03379-4

**Published:** 2024-10-03

**Authors:** Yonghua Feng, Shicheng Xu, Yi Feng, Na Zhao, Linan Xu, Ye Fang, Hongen Xu, Lu Mao, Zhigang Wang, Jiancheng Guo, Guiwen Feng, Jia Rao, Wenjun Shang

**Affiliations:** 1 Department of Renal Transplantation, The First Affiliated Hospital of Zhengzhou University, Zhengzhou, Henan 450052 China; 2 BGI College & Henan Institute of Medical and Pharmaceutical Sciences, Zhengzhou University, Daxuebei Road No. 40, Zhengzhou, 450052 China; 3 Academy of Medical Sciences, Precision Medicine Center of Zhengzhou University, Zhengzhou University, Zhengzhou, Henan, 450052 China; 4 Department of Nephrology, Children’s Hospital of Fudan University, National Pediatric Medical Center of CHINA, No. 399 Wanyuan Road, Shanghai, 201102 China; 5 Shanghai Key Lab of Birth Defect, Children’s Hospital of Fudan University, Shanghai, China; 6 The Research and Application Center of Precision Medicine, The Second Affiliated Hospital of Zhengzhou University, Zhengzhou, 450000 China; 7 National Key Laboratory of Kidney Diseases, Beijing, 100853 China

**Keywords:** Genetic, Kidney failure, Kidney transplantation, Pediatric

## Abstract

**Background:**

The growing body of research on kidney disease in children has identified a broad spectrum of genetic etiologies.

**Methods:**

We conducted a prospective study to evaluate the efficacy of an optimized genetic test and subclinical changes in a real-world context before kidney transplantation. All cases involved recipients under the age of 18 who underwent whole exome sequencing (ES) between 2013 and 2022.

**Results:**

The study population included 244 children, with a median age of 13.1 years at transplantation. ES provided a molecular genetic diagnosis in 114 (46.7%) probands with monogenic variants in 15 known disease-causing genes. ES confirmed the suspected clinical diagnosis in 74/244 (30.3%) cases and revised the pre-exome clinical diagnoses in 40/244 (16.4%) cases. ES also established a specific underlying cause for kidney failure for 19 patients who had previously had an unknown etiology. Genetic diagnosis influenced clinical management in 88 recipients (36.1%), facilitated genetic counseling for 18 families (7.4%), and enabled comprehensive assessment of living donor candidates in 35 cases (14.3%).

**Conclusions:**

Genetic diagnosis provides critical insights into the pathogenesis of kidney disease, optimizes clinical strategies concerning risk assessment of living donors, and enhances disease surveillance of recipients.

**Supplementary Information:**

The online version contains supplementary material available at 10.1186/s13023-024-03379-4.

## Introduction

Kidney transplantation is widely regarded as the most effective treatment option for patients with kidney failure. Due to advancements in surgical techniques, immunosuppression protocols, and clinical management of post-transplant complications, the five-year graft survival rates for kidneys obtained from deceased and living donors have reached 75.3% and 85.3% [1, 2], respectively. The primary causes of kidney failure may vary depending on the patient’s age, medical history, and other factors. Therefore, a comprehensive evaluation and diagnosis by a qualified healthcare professional is essential to determine the most appropriate treatment plan for each patient awaiting transplantation.

In recent years, significantly more attention has been paid to pediatric kidney transplants and their unique characteristics. Based on the clinical phenotype, the most common causes of kidney failure in children and adolescents include glomerulonephritis, cystic ciliopathies/ nephronophthisis (NPHP), tubulopathy, nephrolithiasis/kidney calcinosis, congenital abnormalities of the kidney and urinary tract (CAKUT), and chronic kidney disease (CKD) of unknown origin [3]. However, these rare congenital kidney diseases are prone to being overlooked during the onset of dialysis and at the time of kidney transplantation, as the differential diagnosis of classical phenotypes is often not considered [4]. Clinicians are now becoming more aware of the significant role played by genetic factors in the onset and advancement of certain forms of CKD, especially in individuals with early-onset kidney disease [5, 6]. Mann et al. reported a single-center study showing that 32.7% of pediatric transplant recipients had a genetic cause of CKD [7]. Yishay et al. found a 45% genetic diagnostic rate among Israeli children with kidney failure [8]. A genetic testing study for renal failure in children was also conducted in China [9]. However, these studies were either retrospective post-transplantation studies or focused on kidney failure and dialysis management rather than kidney transplantation.

Genetic sequencing techniques have helped uncover the etiology of CKD and can help predict the progression to kidney failure and the outcomes of transplantation, including allograft rejection [10–13]. Whole exome sequencing (ES) has recently been implemented as a genetic diagnostic tool in clinical medicine, but to date its utility in routine pre-transplantation assessment has not been well-described. This prospective study assessed the efficacy of an optimized genetic test and subclinical changes in a real-world context before kidney transplantation.

## Materials and methods

### Study design and participants

The study population included kidney transplant recipients who were referred to the Organ Transplant Center at the First Affiliated Hospital of Zhengzhou University between January 2013 and December 2022. All participants signed an institutional review board-approved informed consent form before enrollment. Two caregivers or patients opted out of being informed about genetic tests and disclosing any genetic findings. Participants could also choose whether or not to have their samples and data used for future research, either anonymously or not. After obtaining consent, DNA samples were collected and analyzed as part of pre-transplant care. The institutional review board of the First Affiliated Hospital of Zhengzhou University approved and supervised the study protocol (NO. 2013_KY-073). All procedures complied with the Guidance of the Chinese Ministry of Science and Technology (MOST) for the Review and Approval of Human Genetic Resources, which requires formal approval for the export of human genetic material or data from China, and all procedures were conducted in accordance with the Helsinki Declaration.

Eligible patients were under 18 at the time of kidney transplantation; all graft recipients with a functional kidney transplant and at least one valid follow-up visit were included. These patients were referred for the evaluation and management of kidney disease and consented to participation in the general genetic research program. Patients were excluded if they developed kidney failure secondary to kidney disease (e.g., long-term history of diabetes mellitus before kidney failure, systemic lupus erythematosus, acquired obstructive uropathy, tumor, etc.). All participants were registered in the Chinese Scientific Registry of Kidney Transplantation (CSRKT, https://www.csrkt.org.cn/door/index) [14].

### Phenotyping

Upon registration, a genetic counselor generated a three-generation pedigree based on the family history reported by the proband’s parents/guardians. Clinical data and routine laboratory tests were determined using the center’s electronic medical records. Clinical experts in pediatric nephrology identified the phenotype data. Parental health records were unavailable for the study, and a physical examination was not conducted. The questionnaire on family history was completed following the clinical interview between physician and parents.

The primary clinical diagnosis of each patient was determined using a medical history review and referral from a primary nephrologist. Each diagnosis was categorized into one of the following clinical diagnostic categories: (i) nephritis, glomerulopathy presented with hematuria and proteinuria, encompassing membranoproliferative glomerulonephritis, mesangial proliferative glomerulonephritis, crescentic glomerulonephritis, hemolytic uremic syndrome; (ii) steroid-resistant nephrotic syndrome (SRNS), nephropathy with biopsy findings of focal segmental glomerulosclerosis (FSGS); (iii) congenital anomalies of the kidney and urinary tract (CAKUT), defined as any abnormality of number, size, shape, or anatomical position within the kidneys or urinary tract; (iv) cystic nephropathy including nephronophthisis (NPHP), medullary cystic disease, and other kidney cystic ciliopathies; (v) tubulopathy, including clinical diagnosis of tubulopathy and tubulointerstitial nephritis confirmed by kidney biopsy; (vi) nephrolithiasis and kidney calcinosis; or (vii) kidney failure of unknown etiology (KFu).

### Whole exome sequencing

All enrolled patients and their families underwent ES before transplantation. The genomic DNA of all probands and their family members (parents and siblings) was extracted from peripheral blood leukocytes using the GenMagBio Genomic DNA Purification kit following the manufacturer’s instructions (GenMagBio, Changzhou, China). Quality control was performed using agarose gel electrophoresis and a NanoDrop One Spectrophotometer (Thermo Scientific, American) to verify DNA integrity and concentration. DNA was randomly fragmented using a sonicator (Bioruptor^®^ PicoSonication system, Diagenode Belgium) to produce ~ 280 bp genomic fragments. The DNA fragments were end-repaired, and the VAHTS™ Universal DNA Library Kit for MGIEasy (MGI Tech Co., Ltd, Shenzhen, China) was used for library preparation. Exome fragments were captured and enriched using MGI Exome Capture V5 (MGI) following the manufacturer’s protocol. The resulting libraries were sequenced on an MGISEQ-2000RS machine (MGI) to obtain 150 bp paired-end reads at the Precision Medicine Center of Zhengzhou University, Zhengzhou, China.

### Bioinformatics analysis and variant classification

Trimmomatic (version 0.30) was used to remove the adapter sequence and low-quality reads in preparation for data processing. High-quality clean reads were aligned to the human reference genome (version GRCh37) using the Burrow Wheeler Aligner (version 0.7.17-r1188). Duplicate reads were marked using sambamba (version 0.6.8) [16]. Variants, including single-nucleotide variants (SNVs) and insertion-deletions (indels), were identified using the Genome Analysis Toolkit version 4 (GATK4) HaplotypeCaller, and then annotated using Vcfanno with several annotation databases, such as 1000 Genomes Project database, dbSNP, Exome Aggregation Consortium (ExAC), Genome Aggregation Database (gnomAD), ClinVar, InterVar, and dbNSFP. The best practice pipeline based on bcbio-nextgen (https://github.com/bcbio/bcbio-nextgen) was utilized to process all the steps described above. Variants were filtered if the minor allele frequency of the variant was > 5% in the general population, based on at least 2000 alleles observed in the gnomAD database. Exceptions were made for variants listed in the BA1 exception list or pathogenically linked to diseases in the Clinvar database. Candidate variants were interpreted based on the American College of Medical Genetics and Genomics and the Association for Molecular Pathology (ACMG) guidelines by an expert panel of nephrologists, bioinformaticians, and genetic counselors, as previously described [3, 6]. All diagnostic variants were confirmed by Sanger sequencing in the original DNA samples, and when available, were tested for family segregation. Oncoplots and summarized information were then graphed through the Maftools package in the R version 4.0.4.

### Copy number variant (CNV) analysis

For probands with negative ES results for SNVs and indels, we performed CNV analysis of ES data using DECoN (V1.0.2) software with the default setting, a tool with the highest performance evaluated by independent groups [18]. Candidate CNVs identified by DeCoN were further tested for segregation in the family and validated by qPCR.

## Results

### Clinical characteristics

**Fig. 1:**
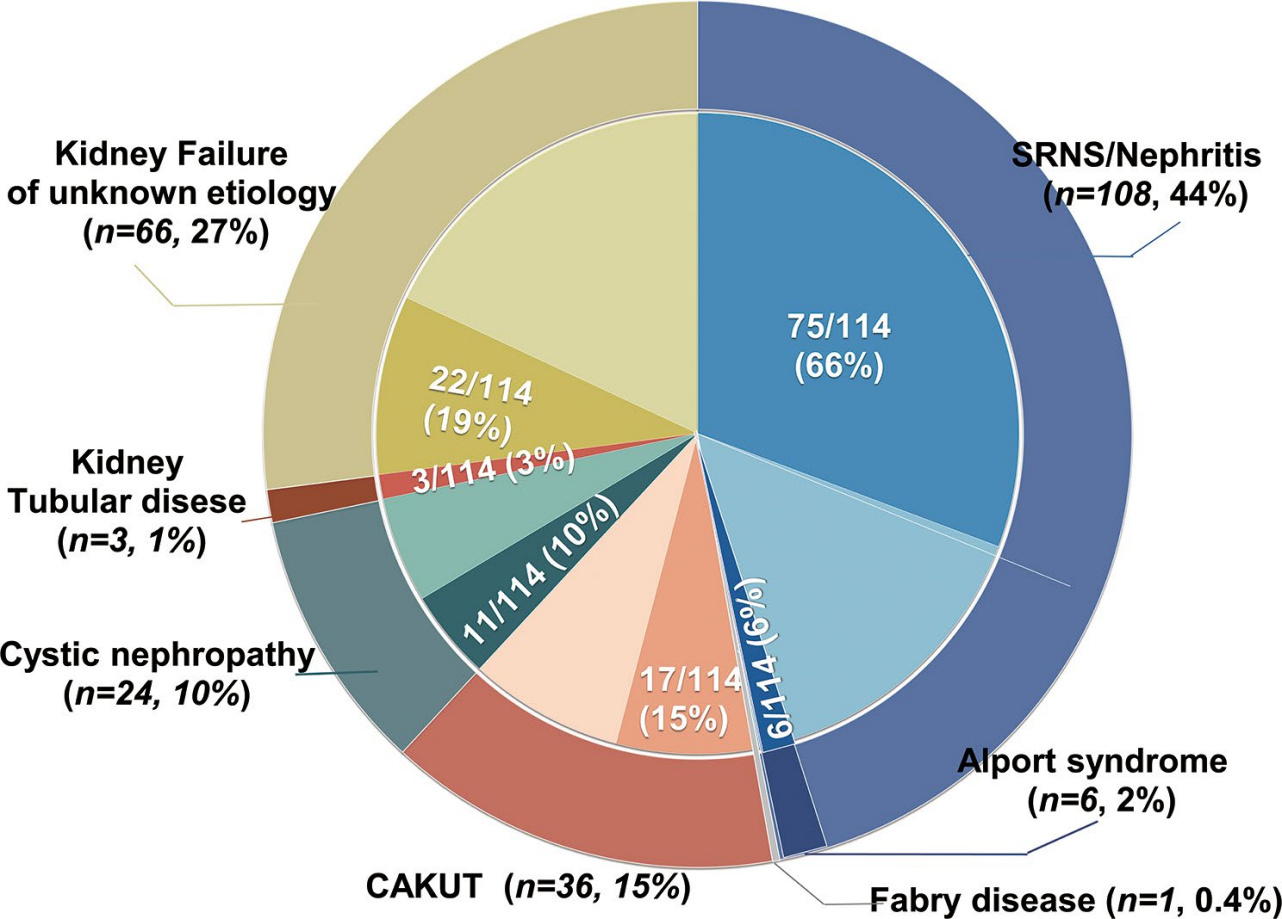
Distribution of clinical diagnosis and post-exome diagnosis in 244 pediatric kidney transplant recipients. The outer circle represents the numbers and percentages of transplant recipients who were classified into one of five clinical diagnostic groups: steroid-resistant nephrotic syndrome (SRNS) or nephritis (process blue), Alport syndrome (satin silver), Fabry disease (navy blue), congenital anomalies of the kidney and urinary tract (CAKUT, dark maroon), kidney cystic disease (cade blue), tubulopathy (saddle brown), and kidney failure of unknown etiology (KFu, pale goldenrod). Inner segments represent for each diagnosis group the relative fraction of patients in whom a final gene diagnosis was confirmed post exome sequencing (dark color) or the unsolved patients in whom pathogenic variants in monogenetic disease-causative genes were identified (light color)

In total, 254 children were included in the kidney transplantation registry from 2013 to 2022. Of these, 244 (males: females 1.6:1) enrolled in the study with written consent. All 244 patients received first-time graft transplantation, at a median age of 13.1 years. They were followed for a median of 2.2 years (interquartile range [IQR], 1.5–4.7 years), resulting in 791.6 person-years of follow-up. The majority of this time (657 years, 97.5%) was spent with a functioning transplant. Phenotypic profiling revealed that the initial clinical diagnoses included SRNS/nephritis (108/244, 44.2%), CAKUT (36/244, 14.8%), kidney cystic disease (24/244, 9.8%), Alport syndrome (6/244, 2.5%), tubulopathy (3/244, 1.2%), Fabry disease (1/244,0.4), and kidney failure of unknown etiology (KFu, 66/244, 27.0%) (Fig. 1). Biopsy-based diagnosis was registered in 20 patients. Extrarenal phenotypes were observed in 52 patients, including hearing loss (*n* = 16), cardiological disorders (*n* = 6), neurological disorders (*n* = 5), visual loss (*n* = 3), achromatopsia (*n* = 1), nystagmus (*n* = 1), and short stature (*n* = 21). In total, 18 probands had a family history of kidney disorders.

### A monogenic cause is identified in 46.7% of kidney transplant recipients

ES was conducted for all families with the exception of 19; parental samples were unavailable for one of these 19 families. The average sequencing depth for all samples was 146X (range 97–215). More than 95% of designed exonic regions were sequenced 20 times for all samples (QC metrics for all samples are provided in Supplemental Table 1). No instances of consanguinity were observed in any of the families. Known variants of pathogenic genes from the registration records were confirmed in 58 cases. None of the variants that had been diagnosed previously were missed with sequencing.

**Fig. 2:**
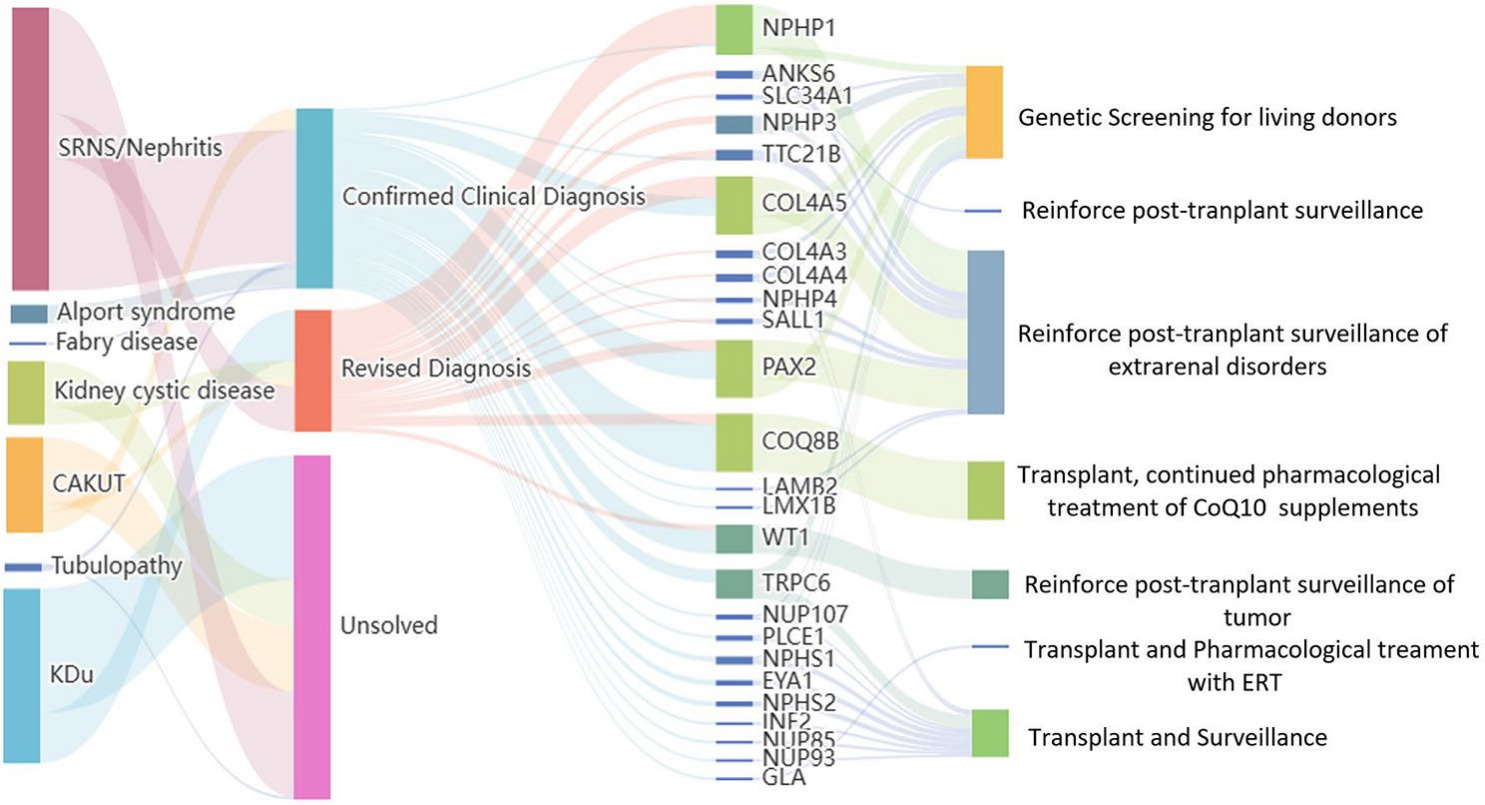
Landscape of the frequency of genes and mutation patterns identified in the pediatric kidney transplant cohort. An oncoplot shows all of the disease-causative genes across our cohort of 114 children with kidney failure. Mutation types and frequencies are summarized for each gene on the right and the mutational burden for each case is shown at the top

**Fig. 3:**
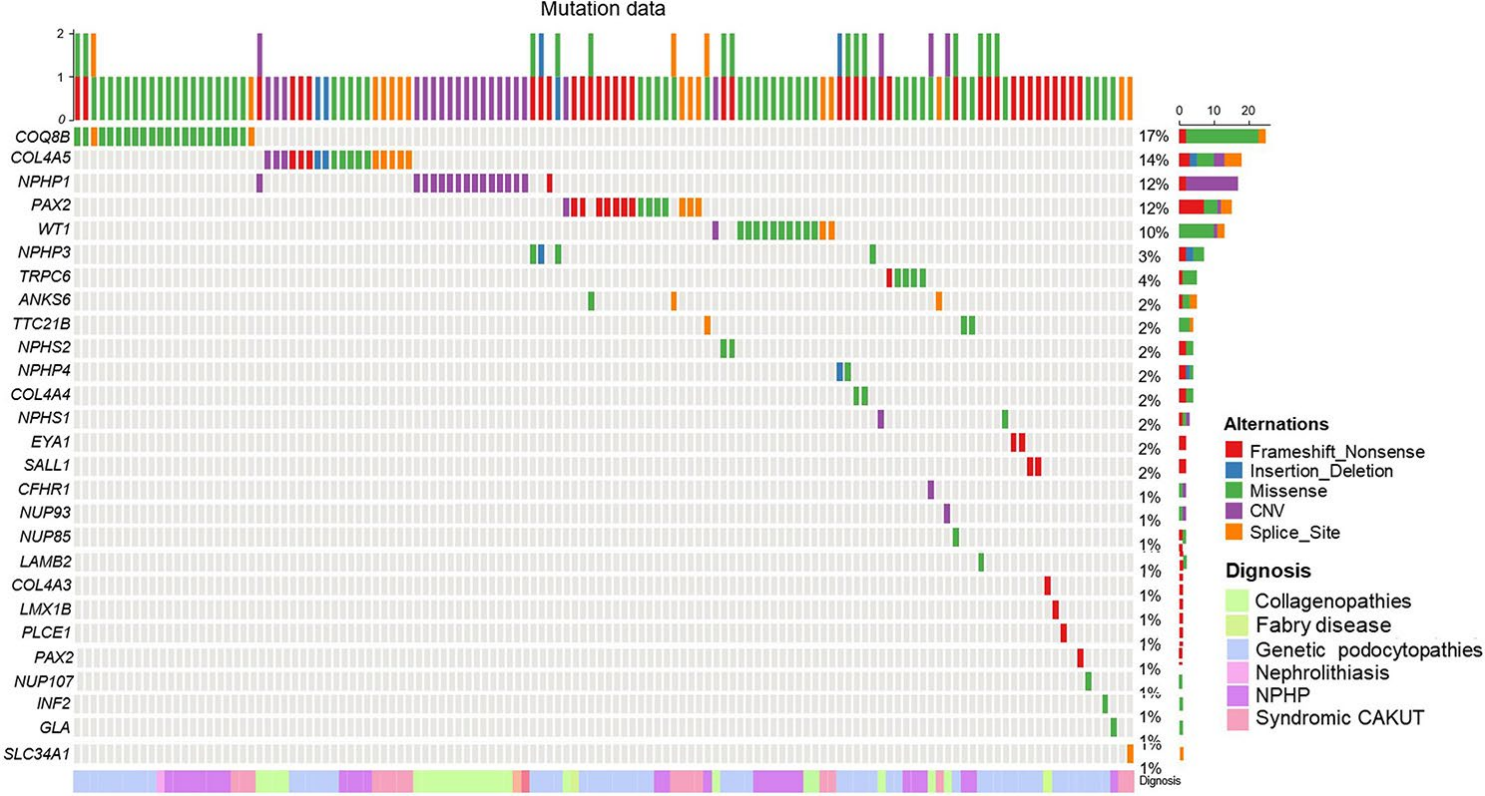
Sankey diagram of the trajectories between initial clinical diagnoses, genetic diagnosis, disease-causative genes, and clinical implementation. Left and middle: division of the initial clinical diagnosis and post-exome diagnosis and monogenic disease-causative genes. Middle and right: genetic diagnosis and clinical implementation. The width of the lines in the Sankey plot is proportional to the relative quantity of cases within each group

ES provided a molecular genetic diagnosis for the 114 (46.7%) probands with monogenic variants in 15 known disease-causing genes. Pathogenic or likely pathogenic variants of six genes accounted for 36 (16.1%) patients with autosomal dominant diseases, 19 genes accounted for 62 (25.4%) patients with autosomal recessive diseases, and two genes accounted for 16 (6.6%) genetic diagnoses of X-linked recessive diseases (Supplemental Table 2). Of the 115 diagnosed pathogenic or likely pathogenic variants, 52 were missense, 34 were frameshift or nonsense, eight were CNVs, 19 were canonical splice-site variations, and two were indels (Fig. 2). In 7% of the probands (17/244), we detected variants of uncertain significance in a gene known to cause kidney disease (Supplemental Table 3). The five genes *COL4A5*, *COQ8B*, *NPHP1*, *PAX2*, and *WT1* accounted for 66.9% of the monogenetic kidney disease diagnoses (Fig. 3).

### Providing a precise etiologic diagnosis for kidney transplant recipients

The percentages of patients for whom we established a molecular genetic diagnosis varied across the clinical diagnostic groups (Fig. 1). ES confirmed the suspected clinical diagnosis in 74/244 (30.3%) of cases and revised the pre-exome clinical diagnoses in 40/244 (16.4%) of cases, including establishing a specific underlying cause for kidney failure in 19 patients with KFu (Fig. 2; Table 1).

**Table 1:** Summary of clinical utility in kidney recipients post exome diagnosis

Post-exome diagnosis subgroup (number)	Revised diagnosis	Genetic screening for living donors	Additional workup / Change in surveillance post transplantation	Additional treatment following transplant	Reproductive counseling
Collagenopathies (*n* = 17)	10	11	10	0	5
Fabry disease (*n* = 1)	0	0	1	1	0
Genetic podocytopathies (*n* = 56)	6	10	16	22	7
Nephrolithiasis (*n* = 1)	1	1	1	1	0
NPHP (*n* = 26)	26	7	26	0	4
Syndromic CAKUT (*n* = 13)	5	6	12	0	2

*CAKUT congenital anomalies of the kidney and urinary tract; *NPHP* nephronophthisis

Among the patients with glomerulopathy, including SRNS and nephritis, monogenic podocytopathies were identified in 49 patients (*COQ8B* [*n* = 18], *WT1* [*n* = 9], *PAX2* [*n* = 6], *TRPC6* [*n* = 5], *NPHS1* [[*n* = 2], *NPHS2* [*n* = 2], *PLCE1* [*n* = 1], *NUP107* [*n* = 1], *NUP85* [*n* = 1], *NUP93* [*n* = 1], *LMX1B* [*n* = 1], *LAMB2* [*n* = 1], *INF2* [*n* = 1]). In patients with an a priori clinical diagnosis of glomerulopathy, pathogenic variants in *COL4A5* or *COL4A3* were detected in eight individuals, confirming the diagnosis of Alport syndrome. A family history of nephrosis was reported in all seven families with an Alport syndrome diagnosis. Alport syndrome was identified in an additional seven families who were initially diagnosed with either nephrotic syndrome or nephritis. Three of these families included multiple affected individuals. Genetic findings that modified the diagnosis in 19 patients included mutations in patients with NPHP (*NPHP1* [*n* = 4], *TTC21B* [*n* = 3], *ANKS6* [*n* = 1], *NPHP3* [*n* = 1], *NPHP4* [*n* = 1]), collagenopathies (*COL4A5* [*n* = 7], *COL4A3* [*n* = 1]), and Fabry disease (*GLA* [*n* = 1]).

Among the patients with CAKUT, pathogenic variants were detected in four known disease-causing genes, including *PAX2* (*n* = 5), *EYA1* (*n* = 2), and *SALL1* (*n* = 1). One patient clinically diagnosed with CAKUT had pathogenic variants in the gene *NPHP1*. Pathogenic variants were detected in ciliopathy genes *NPHP1* (*n* = 5) and *NPHP3* (*n* = 2) in the seven patients with cystic kidney disease. For another two patients with an initial diagnosis of tubulopathy, the genetic diagnosis was confirmed with *NPHP1* and *NPHP4*, respectively.

Among the patients who developed kidney failure without a known etiology, we confirmed the monogenetic kidney disorders in 11 known disease-causative genes, including *PAX2* (*n* = 4), *COQ8B* (*n* = 4), *NPHP1* (*n* = 3), *WT1* (*n* = 2), *COL4A5* (*n* = 1), *COL4A4* (*n* = 1), *ANKS6* (*n* = 1), *TTC21B* (*n* = 1), *SLC34A1* (*n* = 1), and *SALL1* (*n* = 1).

### Clinical implementation of genetic diagnosis

It takes us about three weeks to perform the WES and to provide the results to the transplant team. Multidisciplinary team would give a consult for each case before transplant surgery. Genetic testing has three main applications in clinical kidney transplantation: risk assessment of donors and family counseling, identification of combined therapy schemes for recipients with genetic etiology, and improvement of post-transplant surveillance (Table 1; Fig. 2). Supplementary Table 2 provides detailed information.

First, the final molecular diagnosis allowed for genetic counseling of the patients’ family members and a full assessment of the living donor candidates. After obtaining informed consent, genetic screening for living donors was conducted in 35 families (Table 1). Reproductive counseling was also provided for 18 families with confirmed genetic diagnoses.

Second, genetic tests provided crucial information for targeted therapies in 24 recipients, which could affect graft function or survival following transplantation. For example, it was necessary to continue the pharmacological treatment of enzyme replacement therapy (ERT) following transplantation for patients with Fabry disease. This provided the clinical clue to close follow-up of vasculitis problems, such as cardiopathy. For the 22 patients diagnosed with Coenzyme Q10 (CoQ10) deficiency-associated glomerulopathy caused by the pathogenic variants of *COQ8B*, oral supplementation with CoQ10 should be continued following transplantation. And one case with the pathogenic variant of *SLC34A1* continued the treatment for osteoporosis.

Third, genetic analysis improved post-transplantation surveillance for 41 children (Supplementary Table 2). In cases with a final diagnosis of syndromic kidney disease (*PAX2*, *EVA1*, *SALL1*, *NPHP1*, *NPHP3*, *NPHP4*, *TTC21B*, *ANKS6*, *COL4A5*, *COL4A4*), more details were added to the surveillance program, including ophthalmological, otorhinolaryngological, and psychomotor development evaluations during childhood and adolescence. Among the seven patients with identified *COL4A5* variants who were initially diagnosed of FSGS, three children developed into KF without hearing impairment or ophthalmological abnormalities. These three patients with variants in *COL4A5* (p.Gly435Ar; p.Pro856GlnfsTer19; p.Gly51Arg) need further surveillance for hearing or vision problems as well. For the 11 cases diagnosed with *WT1*-related nephropathy, the decision to perform prophylactic nephrectomy was based on the genetic identification of *WT1* mutations supporting the potential risk of malignancy. The median age of prophylactic nephrectomy was 9.3 years old (IQR, 5.7–13.5 years old). Cancer surveillance was routinely conducted in these patients following transplantation. No complications after nephrectomy was reported.

We emphasize the role of surveillance in cases even without a definitive molecular diagnosis, such as the recurrence of FSGS after kidney transplantation. In the 40 patients (34.8%) referred for FSGS or SRNS, the genetic diagnosis failed to establish this, which could indicate a high risk of post-transplantation recurrence. Nonetheless, among the 75 patients with a definitive molecular diagnosis for SRNS or glomerulopathy, there were no reported cases of proteinuria recurrence during the median follow-up of 2.0 years after kidney transplantation.

## Discussion

In this prospective study, we comprehensively evaluated the clinical utility of pre-transplant genetic testing in pediatric kidney transplant recipients. Through trio-ES, we achieved a high diagnostic yield of 46.7% in a cohort of 244 children. The genetic findings facilitated individualized care for transplant recipients.

Several previous studies have used genetic testing for patients with CKD, achieving diagnostic rates ranging from 24 to 61% for adults [6, 19, 20] and 33–45% for children [3, 7–9]. The dissimilarities in diagnostic yield between these studies likely resulted from differences in sample size, inclusion criteria, and sequencing approaches. In some studies, a higher genetic yield was associated with a younger proband age when the diagnostic ratio in the pediatric cohort was compared to that in the adult cohort [6–8]. Despite the current recommendations for genetic counseling and screening for most children with kidney failure [20, 21], our findings indicate that this clinical practice has often been overlooked: pre-transplant genetic testing was conducted for only 23.8% of the recipients. Previous research has demonstrated that establishing a genetic diagnosis has a beneficial impact on the clinical management of kidney transplantation [9, 10]. Our genetic findings helped inform kidney donor selection; they also helped characterize the nature of disease in each recipient and informed post-transplantation surveillance.

Kidney transplantation has been successfully conducted in pediatric cohorts, and graft survival has markedly improved over the past decades [17, 22]. Many factors influence kidney allograft function, including donor factors, recipient factors, graft function during transplantation, and immunosuppression effects [15]. It is worth noting that genetic information for target therapies can affect graft survival following transplantation. It is common for a diagnosis of rare kidney disease to be delayed or even missed entirely, which is probably an underrecognized cause of graft dysfunction. Our findings revealed that a timely diagnosis of Fabry disease is critical for transplantation. With regard to CoQ10 deficiency-associated glomerulopathy, oral supplementation with CoQ10 is recommended in kidney transplant recipients for extrarenal symptoms [14, 23, 24]. A recent multicenter study reported that the founder mutations of *COQ8B* led to regional variations in the incidence of CoQ10 deficiency-associated glomerulopathy, and confirmed the geographical clustering of the recurrent variants of *COQ8B* in China [25]. Therefore, prioritizing the genetic screening for CoQ10 deficiency and continuous supplement of CoQ10 in kidney recipients is crucial.

Recurrence of native kidney disease is a well-recognized cause of graft loss [26]. However, the reported recurrence rates vary from 2.6 to 50%, depending on the primary disease, and the likelihood of recurrence increases with time after transplantation [26–28]. Another point to be noted is that only a fraction of the native kidneys underwent a biopsy for confirmation of the cause of kidney failure [16]. Data from France indicate that 20% of biopsies provided an inconclusive diagnosis [23]. In the present study, less than one-tenth of the patients had biopsy-based diagnoses.

About one-fifth of the pediatric patients in our transplantation center registry had an initial diagnosis of undetermined kidney disease. The underdiagnosis of Alport syndrome in 11 cases due to the lack of commercial antibody for collagen stating in histopathological detection and poor quality on electron microscope for renal biopsy. Genetic testing validated the final molecular diagnosis in about half of our cohort, including revised diagnoses in 23% of cases. It is noteworthy that patients with a final diagnosis of monogenetic FSGS or collagenopathy had no recurrence of proteinuria during the follow-up period after transplantation. Our findings suggest that recipients with monogenetic FSGS or collagenopathy are at reduced risk for recurrence, which may result in tailored post-transplantation care. However, considering the high risk of recurrence in individuals with idiopathic FSGS, preemptive plasmapheresis with or without rituximab should be discussed for patients with nephrosis when no pathogenic variants are detected in disease-causative genes.

Genetic diagnosis motivated more individualized care during post-transplantation surveillance. In our pediatric cohort, genetic diagnosis delineated the disease involvement of other organ systems, which helped to promote ophthalmological and otorhinolaryngological psychomotor development evaluations. Cancer surveillance was performed in patients with *WT1* gene mutations because malignancy remains a significant concern for the long-term outcomes of transplantation [15]. There always is room for improve the health related quality of life after kidney transplantation especially in children. Personalized treatment combined with genetic information are needed to optimize the health related quality of life in kidney transplant care.

Our study had several limitations. First, it included a modest cohort size of relative ethnic homogeneity, which could have resulted in selection bias. The primary causes of kidney failure in children vary depending on population characteristics and ethnicity. In our study, SRNS or nephritis was clinically diagnosed in 47% of pediatric recipients and 25% of CAKUT or kidney cystic disease. Based on data from the NAPRTCS registry, the most common diagnosis is CAKUT, which affects 40% of pediatric patients [1]. The pediatric recipients in our center comprised 13.5% of the total number of pediatric kidney transplants from the Chinese Scientific Registry of Kidney Transplantation (CSRKT, https://www.csrkt.org.cn/door/index) [14]. Second, we did not perform a chromosomal microarray-based copy-number variation (CNV) analysis, so we may have missed the detection of microdeletion syndromes. However, we could still detect medium-sized gene deletions in 20 individuals based on the exome data. Third, ES may have led to missing variants in introns and promotor regions, specific CNVs, and variants in exons with low coverage. However, given the progressively declining costs of ES and its utility, as demonstrated in many clinical scenarios [24, 29], it is becoming an efficient and cost-effective diagnostic tool for pediatric kidney transplant recipients.

## Conclusions

The clinical utility of genetic diagnosis can offer validated insights into the underlying causes of kidney disease. This, in turn, can guide clinical decision-making regarding risk assessment of living donors and disease surveillance of recipients, ultimately improving individualized care during kidney transplantation.

## Electronic supplementary material

Below is the link to the electronic supplementary material.

Supplementary Material 1

## Data Availability

The corresponding author had full access to the dataset used and analyzed during the current study. The datasets used during the current study are available from the corresponding author upon reasonable request. The variants have been submitted to ClinVar (https://www.ncbi.nlm.nih.gov/clinvar/, SCV004218419-SCV004218510).
