# Supplementary material for: Genetic testing in pediatric kidney transplant recipients to promote informed choice and improve individualized monitoring
Source: Orphanet J Rare Dis. 2024 Oct 3;19:366. doi: 10.1186/s13023-024-03379-4 (PMC11448020; doi:10.1186/s13023-024-03379-4)
Supplement: Supplementary file 1 — Supplementary Material 1 [file 13023_2024_3379_MOESM1_ESM.pdf]

Table S1: Sample Qulaity Control

Sample	Datasize	Q30	GC_conte	IS_avg	IS_sd	Mapping_	Dup_rate	Avg	Cov1X	Cov10X	Cov20X	Cov50X	Cov100X	Uniformi	target_rate
2080-000	17.82	0.935	0.43	292.4	55	1	0.112	156.7	0.995	0.983	0.966	0.84	0.559	0.929	0.787
100013-0	16.57	0.933	0.44	283.9	53.5	1	0.14	140.8	0.995	0.981	0.963	0.826	0.524	0.935	0.787
2101-000	18.22	0.934	0.43	297.1	56.3	1	0.098	161.6	0.995	0.984	0.967	0.846	0.571	0.928	0.782
210912-0	15.13	0.898	0.44	282.7	48.7	0.999	0.061	139.4	0.994	0.982	0.963	0.813	0.507	0.936	0.779
100103-0	16.02	0.935	0.44	275.1	51.8	1	0.123	138.9	0.994	0.979	0.96	0.823	0.52	0.936	0.785
100217-0	16.09	0.935	0.44	260.7	48.7	0.999	0.122	118	0.995	0.978	0.953	0.763	0.439	0.937	0.662
100007-0	15.14	0.934	0.45	279.4	52.3	1	0.174	122.6	0.994	0.98	0.959	0.8	0.476	0.943	0.793
100105-0	14.25	0.94	0.45	261.8	48.6	1	0.121	125.9	0.993	0.978	0.954	0.781	0.469	0.93	0.795
2102-000	17.61	0.935	0.44	296.6	56.3	1	0.102	156.9	0.995	0.984	0.968	0.85	0.567	0.935	0.787
2079-000	22.18	0.936	0.43	292.4	53.7	1	0.117	193.4	0.995	0.985	0.973	0.888	0.654	0.929	0.787
2105-000	19.07	0.935	0.43	296.4	54.9	1	0.107	167.9	0.995	0.984	0.969	0.858	0.591	0.93	0.785
3010051	14.4	0.885	0.43	294.9	53.2	0.998	0.063	132.5	0.994	0.981	0.961	0.8	0.488	0.936	0.783
3010052	13.74	0.887	0.44	290.7	52.1	0.999	0.058	126.7	0.995	0.982	0.96	0.79	0.466	0.939	0.781
3010079	14.69	0.89	0.44	296	54	0.999	0.061	135.5	0.994	0.982	0.964	0.814	0.5	0.938	0.783
3010111	13.74	0.872	0.49	311.8	59.9	0.999	0.09	142.7	0.998	0.993	0.986	0.932	0.658	0.978	0.764
2087-000	16.88	0.935	0.43	295.6	54.5	1	0.09	151.8	0.995	0.982	0.963	0.83	0.543	0.927	0.785
100009-0	12.23	0.898	0.49	299.4	49.7	0.998	0.061	131.2	0.996	0.99	0.983	0.92	0.603	0.976	0.755
100008-0	15.78	0.937	0.44	268.6	48.9	1	0.162	131.9	0.994	0.98	0.959	0.809	0.502	0.936	0.799
2085-000	18.23	0.934	0.43	293.7	54.3	1	0.092	164.1	0.994	0.982	0.966	0.85	0.58	0.929	0.786
2093-000	16.15	0.93	0.43	313.7	57.8	1	0.102	141.4	0.994	0.979	0.955	0.801	0.51	0.921	0.777
100020-0	14.69	0.929	0.44	294.3	58.4	1	0.12	127.5	0.994	0.979	0.959	0.8	0.477	0.939	0.785
100043-0	13.51	0.937	0.45	261	48.5	1	0.132	116.9	0.993	0.978	0.952	0.764	0.441	0.935	0.794
2114-000	13.74	0.882	0.44	294.7	53	0.999	0.054	120.5	0.995	0.981	0.957	0.775	0.442	0.938	0.743
210909-0	16.05	0.895	0.44	281.3	52.3	0.999	0.055	132.4	0.996	0.983	0.965	0.81	0.489	0.942	0.697
3010110	14.17	0.869	0.48	300	50.8	0.998	0.059	146.2	0.998	0.987	0.977	0.928	0.687	0.966	0.739
210904-0	15.08	0.891	0.44	287.1	49.2	0.999	0.058	139.4	0.994	0.982	0.964	0.817	0.508	0.938	0.78
2081-000	19.14	0.937	0.43	288.2	54.2	1	0.124	165.8	0.994	0.981	0.964	0.84	0.574	0.919	0.785
2066-000	15.28	0.922	0.44	261.9	49.5	1	0.143	132.6	0.994	0.98	0.957	0.792	0.488	0.931	0.788
210922-0	16.95	0.947	0.44	275.8	51.8	0.999	0.081	155.2	0.994	0.982	0.966	0.843	0.56	0.931	0.788
100101-0	12.5	0.954	0.43	268.4	50.9	0.999	0.05	128.6	0.996	0.982	0.96	0.788	0.47	0.939	0.663
100104-0	14.61	0.937	0.44	266.3	48.9	1	0.115	129.6	0.994	0.979	0.956	0.792	0.481	0.934	0.792
100091-0	17.51	0.922	0.49	259.6	101.3	0.998	0.246	99.1	0.993	0.975	0.955	0.78	0.363	0.955	0.511
100106-0	14.41	0.891	0.44	298.5	59.2	0.999	0.058	139.9	0.995	0.984	0.966	0.822	0.512	0.941	0.774
100096-0	14.68	0.936	0.44	277.7	51.9	1	0.11	129.4	0.993	0.978	0.957	0.801	0.486	0.938	0.786
210938-0	19.09	0.947	0.44	275.5	51.4	0.999	0.085	173.4	0.995	0.984	0.971	0.875	0.615	0.935	0.786
100045-0	13.34	0.936	0.45	272.9	53.6	1	0.142	111	0.994	0.978	0.952	0.754	0.417	0.939	0.777
100108-0	13.79	0.944	0.45	279.9	51	1	0.06	140.1	0.994	0.983	0.965	0.828	0.525	0.937	0.79
2075-000	19.06	0.947	0.43	260.3	55.1	1	0.127	150.7	0.995	0.978	0.961	0.84	0.56	0.931	0.733
100023-0	14.92	0.933	0.44	283.4	54.7	1	0.127	128.9	0.994	0.979	0.958	0.797	0.481	0.937	0.788
2092-000	19.71	0.932	0.43	301.6	55.5	1	0.091	175.9	0.995	0.984	0.97	0.873	0.618	0.93	0.782
2096-000	17.96	0.929	0.43	309.5	57.4	1	0.102	158.1	0.994	0.981	0.962	0.835	0.562	0.924	0.781
2077-000	16.89	0.949	0.43	258.5	50.3	1	0.158	139.6	0.993	0.975	0.955	0.815	0.526	0.93	0.796
2064-000	12.63	0.944	0.44	277.6	51.1	0.999	0.106	203.4	0.995	0.985	0.976	0.91	0.692	0.938	0.786
2065-000	12.08	0.885	0.44	294.8	53	0.999	0.059	136.7	0.995	0.983	0.964	0.814	0.502	0.938	0.731
210907-0	18.74	0.947	0.44	269.5	49.8	0.999	0.111	165.1	0.994	0.983	0.97	0.871	0.605	0.937	0.79
210916-0	19.59	0.932	0.44	290.1	53.5	0.999	0.095	175.3	0.994	0.983	0.97	0.878	0.624	0.933	0.786
210939-0	24.43	0.947	0.44	277.8	50.6	0.999	0.109	215.7	0.995	0.985	0.977	0.921	0.721	0.937	0.787
3010109	11.33	0.875	0.48	292	48.6	0.999	0.052	127	0.998	0.991	0.983	0.915	0.598	0.977	0.792
3010117	13.79	0.898	0.49	291.9	48.8	0.998	0.068	143.2	0.998	0.993	0.987	0.935	0.661	0.979	0.745
3010118	15.79	0.902	0.49	284.8	47.2	0.999	0.072	164.8	0.999	0.994	0.989	0.956	0.757	0.979	0.751
3010120	15.95	0.905	0.49	284.5	46.6	0.998	0.084	145.7	0.996	0.991	0.986	0.939	0.677	0.977	0.67
3010133	16.34	0.88	0.44	301.7	56.5	0.999	0.06	125.2	0.995	0.982	0.958	0.78	0.457	0.934	0.779
3010141	14.88	0.918	0.44	277.8	52.1	1	0.125	129.5	0.994	0.979	0.956	0.787	0.474	0.933	0
3010143	17.12	0.942	0.43	268.5	50.8	1	0.186	137.9	0.992	0.97	0.951	0.826	0.536	0.929	0.81
3010148	13.21	0.899	0.49	299.7	49.7	0.998	0.06	116.9	0.998	0.992	0.983	0.89	0.518	0.977	0.744
2108-000	13.93	0.887	0.44	301.6	64.6	0.999	0.061	126.6	0.996	0.983	0.961	0.789	0.464	0.939	0.773
2060-000	15.08	0.887	0.44	293.2	53.2	0.999	0.062	128.3	0.994	0.981	0.959	0.787	0.468	0.937	0.759
210918-0	21.59	0.946	0.44	271.8	50.7	0.999	0.086	193.8	0.995	0.985	0.974	0.897	0.668	0.934	0.779
100040-0	14.6	0.936	0.44	272.5	53.3	1	0.135	112.9	0.996	0.979	0.954	0.761	0.424	0.942	0.718
2086-000	16.76	0.934	0.43	295.7	55.1	1	0.091	151.3	0.994	0.981	0.963	0.831	0.543	0.928	0.787
210913-0	15.88	0.891	0.44	287	50.2	0.999	0.062	142.8	0.995	0.983	0.965	0.826	0.522	0.938	0.764
2083-000	18.4	0.937	0.43	292	54.2	1	0.106	162.8	0.995	0.983	0.965	0.839	0.567	0.923	0.784
210930-0	17.76	0.947	0.44	271.4	49.8	0.999	0.081	162.5	0.994	0.983	0.968	0.858	0.584	0.934	0.788
100100-0	11.56	0.89	0.44	296	54	0.999	0.061	135.5	0.994	0.982	0.964	0.814	0.5	0.938	0.783
210926-0	17.97	0.946	0.44	279.8	50.7	0.999	0.083	162	0.994	0.983	0.969	0.859	0.583	0.935	0.778
210911-0	14.5	0.891	0.45	287.5	50.8	0.999	0.055	133.9	0.995	0.983	0.964	0.813	0.495	0.942	0.778
2088-000	11.77	0.887	0.44	290.7	52.1	0.999	0.058	126.7	0.995	0.982	0.96	0.79	0.466	0.939	0.781
100102-0	13.49	0.934	0.44	277.6	51.5	1	0.114	118.2	0.994	0.977	0.95	0.763	0.437	0.935	0.785
2099-000	18.09	0.937	0.43	289.3	53.6	1	0.11	159	0.995	0.981	0.963	0.836	0.561	0.925	0.785
100041-0	19.16	0.909	0.52	247.9	84.7	0.998	0.186	148.3	0.991	0.977	0.965	0.881	0.607	0.946	0.65
100044-0	13.81	0.885	0.43	294.9	53.2	0.998	0.063	132.5	0.994	0.981	0.961	0.8	0.488	0.936	0.783
100029-0	14.92	0.936	0.44	272.9	51.3	1	0.119	131.1	0.994	0.979	0.957	0.796	0.486	0.932	0.79
2073-000	14.86	0.949	0.44	255.4	54.9	1	0.115	130.7	0.994	0.975	0.951	0.785	0.486	0.924	0.794
100046-0	13.6	0.885	0.43	304.4	57.7	0.998	0.065	122.7	0.995	0.98	0.955	0.771	0.45	0.934	0.778
2095-000	17	0.931	0.43	304.4	55.5	1	0.106	149.1	0.995	0.981	0.96	0.821	0.535	0.926	0.783
100030-0	13.04	0.93	0.44	289.8	61.8	1	0.108	113.3	0.995	0.98	0.952	0.755	0.417	0.94	0.776
210931-0	18	0.943	0.44	283.4	52.2	0.999	0.08	163.6	0.995	0.983	0.969	0.859	0.586	0.934	0.784
210925-0	15.88	0.94	0.43	294.9	51.7	0.999	0.075	142.7	0.995	0.982	0.962	0.819	0.518	0.932	0.772

2072-000	16.86	0.946	0.43	262	53.6	1	0.146	141.1	0.994	0.976	0.956	0.819	0.529	0.928	0.792
2084-000	15.31	0.933	0.43	296.1	54.5	1	0.108	134.7	0.994	0.98	0.956	0.786	0.483	0.928	0.782
100046-0	14.64	0.886	0.43	290.9	52.1	0.998	0.053	122.9	0.997	0.982	0.959	0.776	0.449	0.94	0.569
100056-0	12.22	0.889	0.44	285.3	50	0.999	0.062	138.6	0.994	0.982	0.964	0.818	0.509	0.939	0.784
100033-0	13.94	0.934	0.44	276	53	1	0.12	121.8	0.993	0.979	0.957	0.782	0.456	0.939	0.788
2098-000	20.38	0.936	0.43	291.5	54.2	1	0.121	176.5	0.995	0.982	0.966	0.864	0.611	0.923	0.786
210940-0	17.94	0.941	0.44	273.9	49.1	0.999	0.088	162.9	0.994	0.982	0.966	0.852	0.579	0.93	0.788
2089-000	14.45	0.93	0.44	301.1	60.2	1	0.089	129.2	0.995	0.98	0.956	0.787	0.471	0.933	0.781
2091-000	15.64	0.935	0.43	294.3	57.1	1	0.09	140.5	0.994	0.981	0.961	0.812	0.51	0.93	0.783
2094-000	14.14	0.931	0.43	302.9	56.6	1	0.092	126.4	0.994	0.979	0.951	0.768	0.456	0.924	0.783
2111-000	14.44	0.897	0.44	292.2	52	0.999	0.059	133.9	0.995	0.982	0.96	0.8	0.489	0.936	0.785
2100-000	17.25	0.938	0.43	278.1	50.4	1	0.094	157.1	0.994	0.981	0.962	0.83	0.551	0.922	0.794
100022-0	16.06	0.94	0.44	271.5	49.8	1	0.139	138.1	0.994	0.98	0.959	0.81	0.51	0.932	0.796
2103-000	17.92	0.94	0.44	279.8	52.6	1	0.105	159.7	0.995	0.984	0.968	0.853	0.572	0.935	0.79
2063-000	18.06	0.935	0.44	276.2	51.3	1	0.127	135.4	0.994	0.981	0.961	0.804	0.496	0.933	0.789
2062-000	15.08	0.89	0.43	292.9	51.5	0.999	0.067	129.2	0.995	0.981	0.958	0.788	0.473	0.935	0.779
210924-0	15.12	0.942	0.43	272.4	49.1	0.999	0.081	137	0.994	0.979	0.955	0.787	0.488	0.923	0.779
210902-0	23.08	0.944	0.43	287.1	51.4	0.999	0.145	192.7	0.995	0.985	0.975	0.905	0.684	0.939	0.79
210906-0	14.97	0.893	0.45	280.3	49.6	0.999	0.055	138.6	0.994	0.983	0.965	0.823	0.512	0.941	0.778
210914-0	14.3	0.887	0.44	288.2	52.7	0.999	0.052	126.7	0.995	0.982	0.962	0.793	0.466	0.941	0.743
210917-0	19.85	0.947	0.44	272.7	52.5	0.999	0.081	179.2	0.994	0.984	0.972	0.887	0.639	0.939	0.778
210923-0	15.77	0.949	0.44	270.5	50.8	0.999	0.061	133	0.996	0.981	0.958	0.792	0.482	0.932	0.711
210927-0	21.11	0.945	0.44	275.8	50.8	0.999	0.084	191.7	0.995	0.985	0.973	0.893	0.657	0.932	0.785
210928-0	16.39	0.946	0.43	275.8	50.1	0.999	0.082	146.2	0.995	0.981	0.961	0.821	0.527	0.928	0.77
210929-0	20.55	0.943	0.43	280.5	53.6	0.999	0.065	145.9	0.997	0.984	0.968	0.837	0.534	0.939	0.606
210934-0	18.95	0.947	0.43	274.1	49.6	0.999	0.087	172	0.995	0.983	0.969	0.867	0.605	0.93	0.788
210936-0	17.77	0.946	0.44	273.9	51.4	0.999	0.08	163	0.995	0.982	0.966	0.85	0.576	0.929	0.787
2115-000	14.92	0.887	0.44	294.3	54.7	0.998	0.053	127.2	0.996	0.982	0.961	0.793	0.467	0.94	0.721
2067-000	15.7	0.963	0.46	275.2	53	1	0.061	130.6	0.994	0.981	0.958	0.798	0.491	0.931	0.784
2068-000	16.99	0.936	0.43	295.3	55	1	0.097	161.4	0.995	0.983	0.966	0.844	0.573	0.926	0.791
3010131	16.09	0.931	0.42	304.2	57.5	1	0.12	186.1	0.995	0.983	0.968	0.867	0.631	0.918	0.789
3010132	14.73	0.921	0.42	293.2	53.3	1	0.11	156.6	0.995	0.98	0.958	0.816	0.548	0.913	0.793
100001-0	11.99	0.937	0.43	292.5	54.1	1	0.096	143.9	0.994	0.98	0.958	0.803	0.515	0.924	0.791
100005-0	11.74	0.933	0.42	299.3	55	1	0.115	172.1	0.995	0.983	0.966	0.849	0.594	0.919	0.79
100006-0	16.23	0.933	0.44	283	52.7	1	0.164	133.2	0.994	0.981	0.963	0.824	0.512	0.942	0.791
100010-0	18.31	0.938	0.45	269.3	49.5	1	0.168	149.5	0.994	0.982	0.966	0.852	0.57	0.941	0.79
100012-0	16.47	0.938	0.44	270.5	49.6	1	0.162	137.2	0.994	0.981	0.962	0.825	0.524	0.938	0.797
100017-0	12.41	0.964	0.45	266.9	50.2	1	0.07	138.4	0.994	0.982	0.964	0.816	0.512	0.938	0.793
100018-0	13.42	0.932	0.44	278.4	52.6	1	0.118	113.2	0.994	0.978	0.951	0.752	0.418	0.939	0.759
100021-0	14.67	0.935	0.44	267.8	50.3	1	0.137	126.3	0.994	0.978	0.955	0.786	0.471	0.933	0.791
100024-0	18.57	0.931	0.44	278.8	55	0.999	0.111	118.6	0.997	0.98	0.958	0.778	0.441	0.944	0.574
100025-0	13.42	0.934	0.44	282.6	54.1	1	0.118	117.5	0.994	0.978	0.951	0.761	0.433	0.935	0.789
100055-0	13.93	0.937	0.45	264.4	49.7	1	0.12	121.3	0.993	0.978	0.955	0.775	0.453	0.936	0.783
100056-0	12.22	0.936	0.42	296.8	56	1	0.111	172.3	0.995	0.984	0.968	0.855	0.597	0.923	0.789
100057-0	14.15	0.935	0.45	285.1	53.8	1	0.106	125.2	0.993	0.978	0.958	0.797	0.472	0.938	0.786
100066-0	13.86	0.933	0.44	279.9	53.5	1	0.126	119	0.994	0.978	0.954	0.773	0.445	0.94	0.781
100208-0	12.02	0.936	0.43	296.2	55.6	1	0.112	153.2	0.994	0.982	0.964	0.834	0.552	0.93	0.784
2069-000	14.25	0.936	0.43	295.1	55.3	1	0.117	205.3	0.995	0.985	0.975	0.905	0.69	0.931	0.782
2070-000	14.4	0.937	0.43	297.2	55.7	1	0.121	186.6	0.995	0.985	0.972	0.879	0.639	0.926	0.779
2078-000	21.98	0.934	0.43	300.8	58.4	1	0.115	191.3	0.995	0.985	0.973	0.891	0.655	0.93	0.784
2097-000	15.49	0.924	0.43	314.9	79.5	1	0.11	134.1	0.995	0.979	0.953	0.783	0.482	0.925	0.776
2104-000	17.63	0.923	0.43	295.5	54.6	1	0.106	155.5	0.995	0.983	0.964	0.834	0.552	0.925	0.784
2106-000	17.24	0.938	0.43	287.9	53.8	1	0.108	152.5	0.995	0.983	0.965	0.833	0.546	0.93	0.787
2107-000	18.76	0.933	0.43	297.3	56.7	1	0.111	164.2	0.995	0.984	0.969	0.856	0.584	0.933	0.783
2109-000	19.3	0.935	0.43	294.7	54.4	1	0.103	171.9	0.995	0.984	0.969	0.862	0.6	0.928	0.787
2110-000	12.83	0.864	0.44	282.5	46.5	0.998	0.049	124.7	0.993	0.978	0.954	0.772	0.456	0.934	0.788
2112-000	14.11	0.889	0.44	287.7	50.7	0.998	0.057	130.2	0.995	0.981	0.959	0.792	0.475	0.933	0.78
2113-000	25.17	0.941	0.44	287.3	54	0.999	0.147	192.2	0.997	0.987	0.978	0.915	0.697	0.946	0.733
2116-000	24.12	0.944	0.44	290.8	53.5	0.999	0.148	201.2	0.995	0.985	0.976	0.917	0.709	0.941	0.791
2117-000	13.44	0.891	0.44	290.7	50.6	0.999	0.055	125.4	0.994	0.981	0.96	0.789	0.462	0.938	0.785
2118-000	14.31	0.868	0.44	284.9	49.3	0.999	0.054	131.5	0.995	0.982	0.962	0.804	0.484	0.938	0.774
210901-0	14.61	0.886	0.44	292.4	51.1	0.999	0.055	135.7	0.995	0.982	0.963	0.814	0.501	0.937	0.784
210903-0	15.42	0.896	0.44	283.6	48.8	0.999	0.059	143.8	0.995	0.982	0.964	0.825	0.524	0.936	0.788
210905-0	13.28	0.889	0.44	291.3	50.7	0.998	0.054	122.9	0.994	0.979	0.954	0.764	0.444	0.933	0.776
210910-0	22.06	0.947	0.44	281.2	49.9	0.999	0.134	187.8	0.995	0.985	0.975	0.905	0.677	0.942	0.791
210915-0	22.92	0.939	0.44	295.1	53.4	0.999	0.11	199.6	0.995	0.985	0.975	0.905	0.687	0.935	0.782
210921-0	20.73	0.948	0.44	273.8	51.7	0.999	0.124	177.6	0.995	0.984	0.972	0.881	0.632	0.935	0.785
210933-0	17.61	0.948	0.44	269.3	49.7	0.999	0.09	159.6	0.995	0.983	0.967	0.853	0.578	0.935	0.789
210937-0	17.91	0.946	0.43	275.2	49.6	0.999	0.078	160.1	0.995	0.982	0.965	0.841	0.564	0.925	0.768
210908-0	20.62	0.949	0.44	273.4	50	0.999	0.121	177.2	0.995	0.984	0.972	0.887	0.64	0.938	0.785
210935-0	21.03	0.949	0.44	264.8	46.9	0.999	0.091	189.9	0.994	0.984	0.973	0.895	0.659	0.936	0.785
2090-000	17.76	0.934	0.43	299.9	56.1	1	0.083	159.8	0.995	0.982	0.965	0.843	0.566	0.929	0.78
3010157	16.06	0.893	0.44	283.5	51.1	0.998	0.061	134.4	0.994	0.981	0.957	0.784	0.48	0.929	0.779
3010119	14.97	0.905	0.49	290.6	48.4	0.998	0.088	140.3	0.996	0.991	0.985	0.938	0.663	0.978	0.695
3010167	13.67	0.887	0.44	295.2	51.9	0.999	0.092	120.9	0.995	0.98	0.955	0.765	0.435	0.934	0.777
3010162	14.75	0.864	0.44	283.7	47	0.999	0.063	136.6	0.993	0.976	0.952	0.788	0.492	0.921	0.784
3010116	12.19	0.948	0.5	230.2	49	0.999	0.346	102.6	0.989	0.977	0.954	0.781	0.363	0.95	0.782
3010161	13.78	0.939	0.44	259	49.4	1	0.127	129.1	0.994	0.978	0.954	0.782	0.476	0.93	0.796
3010151	11.95	0.885	0.44	294.4	52.5	0.999	0.098	190.9	0.995	0.977	0.965	0.903	0.685	0.933	0.753
3010152	12.95	0.895	0.44	287	51.4	0.999	0.111	190	0.996	0.977	0.965	0.9	0.681	0.932	0.739

3010145	17.72	0.94	0.43	269.8	52.2	1	0.169	134.3	0.994	0.97	0.952	0.823	0.524	0.933	0.754
3010115	15.1	0.939	0.44	262.2	48.2	1	0.109	124.8	0.993	0.977	0.952	0.771	0.458	0.932	0.798
3010159	15.3	0.891	0.44	292.1	54.6	0.999	0.059	141.2	0.994	0.982	0.964	0.82	0.515	0.935	0.777
3010165	17.64	0.964	0.44	272.1	49.9	1	0.078	179.9	0.995	0.985	0.972	0.877	0.623	0.933	0.507
3010168	15.39	0.891	0.44	284	48.3	0.999	0.093	139.6	0.994	0.981	0.962	0.809	0.501	0.935	0.792

Sample	sample nanme
Datasize	data size of fastq files
Q30	Q30
GC_conte	GC content of all reads
IS_avg	the average size of insert size
IS_sd	the standard deviation of insert size
Mapping_	the mapping rate of reads to the human genome
Dup_rate	dulplication rate
Avg	average depth
Cov1X	1x coverage
Cov10X	20x coverage
Cov20X	20x coverage
Cov50X	50x coverage
Cov100X	100x coverage
Uniformi	0.2*(average depth) coverage
target_r	percent bases on-target

Table S2. Genetic findings in children with kidney failure waiting for kidney transplantation

ID	Suspected clinical diagnosis	Post-exome diagnoses	Gene; c. Change; p. Change; Segregation (p, m)	Inheritance pattern	gnomAD (All)	gnomAD (EA)	ACMG(HGMD)	Clinical implementation
210911	SRNS/FSGS	Genetic podocytopathies	*INF2* (NM_022489.4)	AD				Transplant
			c.383T>C; p.Leu128Pro; (het; p, NA; m, wt)		0	0	DM; VUS	
210927	Nephritis	Collagenopathies	*COL4A5* (NM_033380.3)	XLD				Transplant and Surveillance of hearing loss
			c.1303G>C; p. Gly435Arg (Hemi; p,wt; m,het)		0	0	VUS;N	
100006	SRNS/FSGS	Genetic podocytopathies	*PAX2* (NM_000278.5)	AD				Transplant, Ophthalmological evaluation
			c.76dup;p.Val26fs (het; p,wt;m,wt)		0	0	LP;DM;PMID;29973660	to identify and address any potential visual problems in probands and other carriers in the family
100008	SRNS/FSGS	Genetic podocytopathies	*PAX2* (NM_000278.5)	AD				Transplant, Ophthalmological evaluation
			c.76dup;p.Val26fs (p,wt;m,wt)		0	0	P;DM;PMID;8589702	to identify and address any potential visual problems in probands and other carriers in the family
100010	SRNS/FSGS	Collagenopathies	*COL4A3* (NM_000091.5)	AR				Transplant and Surveillance
			c.1038T>A;p.Tyr346Ter (het; p,het; m,wt)		0	0	LP;DM;PMID;22887978	
			c.1274C>A;p.Ser425Ter (het; p,wt; m,het)		0	0	LP(PVS1,PM2_p);N	
100017	SRNS/FSGS	Genetic podocytopathies	*COQ8B* (NM_024876.4)	AR				Transplant, continued pharmacological
			c.737G>A;p.Ser246Asn (het; p,het;m,wt)		0.00006401	0.0009	P;DM;PMID;28204945	treatment of CoQ10 supplements
			c.1468C>T;p.Arg490Cys (het; p,wt;m,het)		0.000060045	0.00082	P(PM2_p,PM3 verystrong,PP1	
100025	SRNS/FSGS	Genetic podocytopathies	*PAX2* (NM_000278.5)	AD				Transplant, Ophthalmological evaluation to
			c.213-8C>A (het; p,wt;m,wt)		0	0	LP;N	identify and address any potential visual problems in probands and other carriers in the family
100040	SRNS/FSGS	Genetic podocytopathies	*COQ8B* (NM_024876.4)	AR				Transplant, continued pharmacological
			c.737G>A;p.Ser246Asn (het; p,het;m,wt)		0.00006401	0.0009	P;DM;PMID;28204945	treatment of CoQ10 supplements
			c.1468C>T;p.Arg490Cys (het; p,wt;m,het)		0.000060045	0.00082	P(PM2_p,PM3 verystrong,PP1	
100045	SRNS/FSGS	Genetic podocytopathies	*COQ8B* (NM_024876.4)	AR				Transplant, continued pharmacological
			c.737G>A;p.Ser246Asn (HOM;p,het;m,het)		0.00006401	0.0009	P;DM;PMID;28204945	treatment of CoQ10 supplements
100055	SRNS/FSGS	Genetic podocytopathies	*TRPC6* (NM_004621.6)	AD				Transplant and Surveillance
			c.991G>A;p.Gly331Arg (het; p,wt;m,wt)		0	0	LP;N	
100104	Nephritis	Collagenopathies	*COL4A5* (NM_033380.3)	XLD				Transplant and Surveillance of hearing loss
			c.1117C>T;p.Arg373Ter (Hemi; p,wt;m,het)		0	0	P;DM;PMID;8651296	
2060	SRNS/FSGS	Genetic podocytopathies	*COQ8B* (NM_024876.4)	AR				Transplant, continued pharmacological
			c.1468C>T;p.Arg490Cys (het; p,het;m,wt)		0.000060045	0.00082	P;DM;PMID;28204945	treatment of CoQ10 supplements
			c.737G>A;p.Ser246Asn (het; p,wt;m,het)		0.00006401	0.0009	P(PM2_p,PM3_verystrong,PP1,P	
2061	SRNS/FSGS	Genetic podocytopathies	*NPHS2* (NM_014625.4)	AR				Transplant and Surveillance
			c.9971C>T;p.Ser313Leu (het;p,het;m,wt)		0	0	VUS;N	
			c.467dup;p.Leu56Phefs (het;p,wt;m,het)		0	0	P(PVS1,PM2_p,PP4);	
2064	SRNS/FSGS	Genetic podocytopathies	*NPHS2* (NM_014625.4)	AR				Transplant and Surveillance
			c.871C>T;p.Arg291Trp (het;p,het;m,wt)		0.000031863	0	VUS;DM; PMID:10742096	
			c.467dup;p.Leu156fs (het;p,wt;m,het)		0.00064185	0	P(PVS1,PM2_p,PP4);	
2071	SRNS/FSGS	Genetic podocytopathies	*WT1* (NM_024426.6)	AD				Transplant and Surveillance of tumor
			c.1384C>T;p.Arg462Trp (p,wt;m,wt)		0	0	LP;DM;PMID;18516627	
2077	Alport syndrome	Collagenopathies	*COL4A5* (NM_033380.3)	XLD				Transplant and Surveillance
			c.4528+1G>C (Hemi; p,wt;m,het)		0	0	P;N	
2080	SRNS/FSGS	Genetic podocytopathies	*COQ8B* (NM_024876.4)	AR				Transplant, continued pharmacological
			c.532C>T;p.Arg178Trp (het;p,het;m,wt)		0.000024216	0.00016	LP;DM;PMID;24270420	treatment of CoQ10 supplements
			c.737G>A;p.Ser246Asn (het;p,wt;m,het)		0.00006401	0.0009	P(PM2_p,PM3 verystrong,PP1	
2081	SRNS/FSGS	Genetic podocytopathies	*COQ8B* (NM_024876.4)	AR				Transplant, continued pharmacological
			c.748G>C;p.Asp250His (het;p,het;m,wt)		0.000044126	0	P;DM;PMID;28204945	treatment of CoQ10 supplements
			c.1297-2A>G (het;p,wt;m,het)		0.00059854	0	LP(PVS1,PM2_p);N	
2084	Nephritis	NPHP	*NPHP4* (NM_015102.5)	AR				Transplant, Surveillance of extrarenal
			c.4237_4249del;p.Asp1413fs (het;p,het;m,wt)		0	0	P;N	manifestation including dysplastic phenotype of lvier, eye and central nervous system
			c.3062del;p.Gln1021fs (het;p,wt;m,het)		0	0	LP(PVS1,PM2_p);N	
2085	Nephritis	NPHP	*NPHP1* (NM_001128178.3)	AR				Transplant, Surveillance of extrarenal
			Deletion Exon1-20 (HOM;p,het;m,het)		0	0	P;DM;PMID;29146700	manifestation including dysplastic phenotype of lvier, eye and central nervous system
2087	SRNS/FSGS	Genetic podocytopathies	*COQ8B*(NM_024876.4)	AR				Transplant, continued pharmacological
			c.532C>T;p.Arg178Trp (het;p,NA;m,NA)		0.000024216	0.00016	VUS;DM; PMID:24270420	treatment of CoQ10 supplements
			c.1468C>T;p.Arg490Cys (het;p,NA;m,NA)		0.000058435	0.00018	P(PM2_p,PM3 verystrong,PP1	
2096	SRNS/FSGS	Genetic podocytopathies	*PAX2* (NM_000278.5)	AD				Transplant, Ophthalmological evaluation
			c.76dup;p.Val26fs (het;p,NA;m,NA)		0	0	P;DM;PMID;8589702	to identify and address any potential visual problems in probands and other carriers in the family
2106	SRNS/FSGS	Genetic podocytopathies	*WT1* (NM_024426.6)	AD				Transplant, Surveillance of tumor
			c.1432+4C>T (het;p,wt;m,wt)		0	0	LP;DM;PMID;23302619	
2107	Nephritis	NPHP	*ANKS6* (NM_173551.5)	AR				Transplant, Surveillance of extrarenal
			c.2397+1G>A (het;p,NA;m,NA)		0	0	LP;N	manifestation including dysplastic phenotype of lvier and central nervous system
			c.1618-3C>G (het;p,NA;m,NA)		0	0	VUS(PM2_p,PM3,PP3);	
2113	SRNS/FSGS	Genetic podocytopathies	*COQ8B* (NM_024876.4)	AR				Transplant, continued pharmacological treatment of CoQ10 supplements
			c.737G>A;p.Ser246Asn (het;p,het;m,wt)		0.00006401	0.0009	P;DM;PMID;28204945	
			c.1468C>T;p. Arg490Cys (het;p, wt; m, het)		0.000060045	0.00082	P(PM2_p,PM3_verystrong,PP1,P	
2118	SRNS/FSGS	Genetic podocytopathies	*COQ8B* (NM_024876.4)	AR				Transplant, continued pharmacological
			c.737G>A;p.Ser246Asn (het;p,NA;m,NA)		0.00006401	0.0009	P;DM;PMID;28204945	treatment of CoQ10 supplements
			c.748G>C;p.Asp250His (het;p,NA;m,NA)		0.000044126	0.0006	P(PM2_p,PM3 verystrong,PP3	
210901	Alport syndrome	Collagenopathies	*COL4A5* (NM_033380.3)	XLD				Transplant and Surveillance
			c.4706G>A;p.Arg1569Gln (Hemi;p,wt;m,het)		0	0	LP;N	
210902	Alport syndrome	Collagenopathies	*COL4A5* (NM_033380.3)	XLD				Transplant and Surveillance
			c.276+3A>C (Het;p,wt;m,wt)		0	0	LP;N	
210905	SRNS/FSGS	Genetic podocytopathies	*COQ8B* (NM_024876.4)	AR				Transplant, continued pharmacological
			c.737G>A;p.Ser246Asn (HOM,p,het;m,het)		0.00006401	0.0009	P;DM;PMID;28204945	treatment of CoQ10 supplements
210906	Nephritis	Collagenopathies	*COL4A5* (NM_033380.3)	XLD				Transplant and Surveillance

			c.81+2dup (Hemi;p,NA;m,het)		0	0	P;N	
210925	Alport syndrome	Collagenopathies	*COL4A5* (NM_033380.3)	XLD				Transplant and Surveillance
			c.4822-1G>T (het;p,NA;m,NA)		0	0	P;N	
210926	SRNS/FSGS	Genetic podocytopathies	*COQ8B* (NM_024876.4)	AR				Transplant, continued pharmacological
			c.1465C>T;p.His489Tyr (het;p,NA;m,NA)		0.000013354	0.00012	VUS;N	treatment of CoQ10 supplements
			c.748G>C;p.Asp250His (het;p,NA;m,NA)		0.000044126	0.00012	P(PM2_p,PM3 verystrong,PP3	
210928	SRNS/FSGS	Genetic podocytopathies	*TRPC6* (NM_004621.6)	AD				Transplant and Surveillance
			c.1891_1894del;p.Val631fs (het;p,het;m,NA)		0	0	LP;N	
210935	SRNS/FSGS	Genetic podocytopathies	*LMX1B* (NM_001174147.2)	AD				Transplant and Surveillance
			c.917del;p.Gln306fs (het;p,NA;m,NA)		0	0	P;N	
3010079	Nephritis	NPHP	*TTC21B* (NM_024753.5)	AR				Transplant, Surveillance of extrarenal
			c.262G>T;p.Asp88Tyr (het;p,NA;m,NA)		0	0	VUS;N	manifestation including dysplastic phenotype of lvier, eye and central nervous system
			c.1552T>C;p.Cys518Arg (het;p,NA;m,NA)		0	0	P(PM2_p,PM3 strong,PP1,PP	
3010052	Alport syndrome	Collagenopathies	*COL4A5* (NM_033380.3)	XLD				Transplant and Surveillance
			c.4511-1G>C (Hemi;p,wt;m,het)		0	0	P;N	
3010104	Nephritis	NPHP	*NPHP1* (NM_001128178.3)	AR				Transplant, Surveillance of extrarenal
			c.1039C>T;p.Arg347Ter (het;p,het;m,wt)		0	0	P;DM;PMID;18076122	manifestation including dysplastic phenotype of lvier, eye and CNS
			Deletion Exon1-20 (HOM;p,wt; m,het)					
3010157	SRNS/FSGS	Genetic podocytopathies	*PAX2* (NM_000278.5)	AD				Transplant, Ophthalmological evaluation
			c.263C>G;p.Pro88Arg (het;p,wt;m,wt)		0	0	P;DM;PMID;31027891	to identify and address any potential visual problems in probands and other carriers in the family
3010115	SRNS/FSGS	Genetic podocytopathies	*NUP85* (NM_024844.5)	AR				Transplant and Surveillance
			c.1933C>T;p.Arg645Trp (het;p,NA;m,NA)		0.00003535	0	LP;DM;PMID;30179222	
			c.1880_1881del;p.Ile627fs (het;p,NA;m,NA)		0.000003977	0	P(PVS1,PM2_p,PP4);N	
3010159	SRNS/FSGS	Genetic podocytopathies	*COQ8B* (NM_024876.4)	AR				Transplant, continued pharmacological
			c.1468C>T;p.Arg490Cys (het;p,het;m,wt)		0.000060045	0.00082	P;DM;PMID;28204945	treatment of CoQ10 supplements
			c.748G>C;p.Asp250His (het;p, wt; m,het)		0.000044126	0.0006	P(PM2_p,PM3 verystrong,PP3	
3010112	SRNS/FSGS	Genetic podocytopathies	*WT1* (NM_024426.6)	AD				Transplant, Surveillance of tumor
			c.1399C>T;p.Arg467Trp (het;p,wt;m,wt)		0	0	LP;DM;PMID;1655284	
100278	Nephritis	Collagenopathies	*COL4A5* (NM_033380.3)	XLD				Transplant and Surveillance
			c.2567delC; p.Pro856GlnfsTer19(Hemi;p,wt;m,het)		0	0	P;DM;PMID;30577881	
100286	Fabry disease	Fabry disease	*GLA* (NM_000169.3)	XLD				Transplant and Pharmacological treament
			c.974G>A;p.Gly325Asp (het; p,wt;m,het)		0	0	LP;DM;PMID;15776423	with ERT
100287	SRNS/FSGS	Genetic podocytopathies	*PAX2* (NM_000278.5)	AD				Transplant, Ophthalmological evaluation
			c.383_384del;p.Thr128LysfsTer (het; p,wt;m,wt)		0	0	P;N	to identify and address any potential visual problems in probands and other carriers in the family
100289	SRNS/FSGS	Genetic podocytopathies	*WT1* (NM_024426.6)	AD				Transplant, Surveillance of tumor
			c.896G>A;p.Arg299Phe (het; p,wt;m,wt)		0	0	LP;N	
3010099	SRNS/FSGS	Collagenopathies	*COL4A5* (NM_033380.3)	XLD				Transplant and Surveillance
			c.3078dup;p.Gly1027ArgfsTer (Hemi; p,wt;m,het)		0	0	P;N	
100302	SRNS/FSGS	Genetic podocytopathies	*WT1* (NM_024426.6)	AD				Transplant, Surveillance of tumor
			c.1384C>T;p.Arg462Trp (het; p,wt;m,wt)		0	0	LP;DM;PMID;18516627	
100316	SRNS/FSGS	Genetic podocytopathies	*NUP93* (NM_014669.5)	AR				Transplant and Surveillance
			Deletion:Exon1-6 (het; p,het;m,wt)		0	0	P;N	
			c.1772G>T;p.Gly591Val (het; p,wt;m,het)		0.000228	0.00023	LP(PM3, PS3, PM2, PP3);N	
100323	SRNS/FSGS	Genetic podocytopathies	*COQ8B* (NM_024876.4)	AR				Transplant, continued pharmacologica
			c.1468C>T;p.Arg490Cys (het; p,het;m,wt);		0.000060045	0.00082	P;DM;PMID;28204945	l treatment of CoQ10 supplements
			c.737G>A;p.Ser246Asn (het; p,wt;m,het)		0.00006401	0.0009	P(PM2_p,PM3 verystrong,PP1	
100324	SRNS/FSGS	Genetic podocytopathies	*WT1* (NM_024426.6)	AD				Transplant, Surveillance of tumor
			c.1379T>C;p.Phe460Ser (het;p,wt;m,wt)		0	0	LP;N	
100327	Nephritis	NPHP	*NPHP1* (NM_001128178.3)	AR				Transplant, Surveillance of extrarenal
			Deletion Exon1-20 (HOM;p,het;m,het)		0	0	P;DM;PMID;29146700	manifestation including dysplastic phenotype of lvier, eye and CNS
100332	SRNS/FSGS	Genetic podocytopathies	*COQ8B* (NM_024876.4)	AR				Transplant, continued pharmacological
			c.532C>T;p.Arg178Trp (het;p,het;m,wt);		0.000024216	0.00016	LP;DM;PMID;24270420	treatment of CoQ10 supplements
			c.737G>A;p.Ser246Asn (het;p,wt;m,het)		0.00006401	0.0009	P(PM2_p,PM3 verystrong,PP1	
100333	SRNS/FSGS	Genetic podocytopathies	*PLCE1* (NM_016341.4)	AR				Transplant and Surveillance
			c.5078_c.5079insGAGGAAAAGG;p.Glu1693Efs (het;p,het;m,wt)		0	0	P;N	
			c.1477C>T;p.Arg493Ter (het;p,wt;m,het)		0.00001204	5.60E-05	P(PVS1,PM2_p,PP4);N	
100344	Alport syndrome	Collagenopathies	*COL4A5* (NM_033380.3)	XLD				Transplant and Surveillance
			Deletion:Exon22-37 (Hemi;p,wt;m,wt)		0	0	P;N	
100348	SRNS/FSGS	Genetic podocytopathies	*TRPC6* (NM_004621.6)	AD				Transplant and Surveillance
			c.430G>C;p.Glu144GLn (het;p,wt;m,wt)		0	0	LP;N	
100366	SRNS/FSGS	Genetic podocytopathies	*WT1* (NM_024426.6)	AD				Transplant, Surveillance of tumor
			c.1367T>G;p.Phe456Cys (het;p,wt;m,wt)		0	0	LP;N	
100370	SRNS/FSGS	Genetic podocytopathies	*COQ8B* (NM_024876.4)	AR				Transplant, continued pharmacological
			c.1468C>T;p.Arg490Cys (het;p,het;m,wt)		0.00006004	0.00082	P;DM;PMID;28204945	treatment of CoQ10 supplements
			c.737G>A; p.Ser246Asn; (het;p, wt; m, het)		0.00006401	0.0009	P(PM2_p,PM3 verystrong,PP1	
100373	SRNS/FSGS	Genetic podocytopathies	*WT1* (NM_024426.6)	AD				Transplant, Surveillance of tumor
			c.896C>T;p.Ser299Phe (het;p,wt;m,wt)		0	0	LP;N	
100379	Nephritis	NPHP	*NPHP1* (NM_001128178.3)	AR				Transplant, Surveillance of extrarenal
			Deletion:Exon1-20 (HOM;p,het;m,het)		0	0	P;DM;PMID;29146700	manifestation including dysplastic phenotype of lvier, eye and CNS
100382	SRNS/FSGS	Genetic podocytopathies	*TRPC6* (NM_004621.6)	AD				Transplant and Surveillance
			c.523C>T;p.Arg175Trp (het;p,wt;m,wt)		0	0	LP;DM;PMID;28204945	
3010131	Nephritis	NPHP	*NPHP3* (NM_153240)	AR				Transplant, Surveillance of hepatic
			c.748C>T;p.Gln250Ter (het;p,het;m,wt);		0	0	P;DM;PMID;33323469	function impairment and liver cirrhosis post transplantation
			c.3757C>G;p.Leu1253Val (het;p,wt;m,het)				VUS(PM2_p,PM3,PP3);DM;PMI	

100390	SRNS/FSGS	Genetic podocytopathies	*LAMB2* (NM_002292.4)	AR				Transplant and Surveillance
			c.1405C>T;p.Arg469Ter (het;p,het;m,wt)		0.00001204	0	P;N	
			c.1066T>A;p.Cys356Ser (het;p,wt;m,het)		0	0	VUS(PM2_p,PM 3,PP3);N	
100391	Alport syndrome	Collagenopathies	*COL4A5* (NM_033380.3)	XLD				Transplant and Surveillance
			Deletion:Exon48-50 (Hemi;p,wt;m,het)		0	0	P;N	
100392	SRNS/FSGS	Genetic podocytopathies	*TRPC6* (NM_004621.6)	AD				Transplant and Surveillance
			c.523C>T;p.Arg175Trp (het;p,wt;m,wt)		0	0	LP;DM;PMID;28 204945	
100394	SRNS/FSGS	Genetic podocytopathies	*NPHS1* (NM_004646.4)	AR				Transplant and Surveillance
			c.3478C>T;p.Arg1160Ter (het;p,het;m,wt)		0	0	P;DM;PMID;991 5943	
			Deletion:Exon23-29 (het;p,wt;m,het)				P(PVS1,PM2_p, PP4);N	
100403	SRNS/FSGS	Collagenopathies	*COL4A5* (NM_033380.3)	XLD				Transplant and Surveillance
			c.2567del (Hemi;p,wt;m,het)		0	0	LP;DM;PMID;30 577881	
100404	SRNS/FSGS	Genetic podocytopathies	*TTC21B* (NM_024753.5)	AR				Transplant and Surveillance
			c.752T>G;p.Met251Arg (het;p,het;m,wt)		0	0	LP;DM;PMID;31 208513	
			c.1552T>C;p.Cys518Arg (het;p,wt;m,het)		0	0	P(PM2_p,PM3_ strong,PP1,PP3,	
100408	SRNS/FSGS	Genetic podocytopathies	*COQ8B* (NM_024876.4)	AR				Transplant, continued pharmacological
			c.1084C>T;p.Arg362Ter (het;p,het;m,wt)		0	0	LP;N	treatment of CoQ10 supplements
			c.1430G>A;p.Arg477Gln (het;p,wt;m,het)		0.000004133	0	LP(PM2,PM3 strong,PP3);D	
100005	CAKUT	syndromic CAKUT	*PAX2* (NM_000278.5)	AD				Transplant, Ophthalmological evaluation
			c.92G>C;p.Arg31Pro (het;p,wt;m,wt)		0	0	LP;N	to identify and address any potential visual problems in probands and other carriers in the family
2117	CAKUT	syndromic CAKUT	*EYA1* (NM_000503.6)	AD				Transplant Surveillance of extrarenal
			c.1697del;p.Lys566fs (het;p,NA;m,wt)		0	0	P;N	manifestation
3010152	Nephritis	Collagenopathies	*COL4A5* (NM_033380.3)	XLD				Transplant and Surveillance
			c.151G>A;p.Gly51Arg (Hemi;p,wt;m,het)		0	0	LP;N	
100303	CAKUT	syndromic CAKUT	*PAX2* (NM_000278.5)	AD				Transplant, Ophthalmological evaluation
			c.76dup;p.Val26GLyfsTer (het;p,wt;m,wt)		0	0	P;DM;PMID;858 9702	to identify and address any potential visual problems in probands and other carriers in the family
100305	CAKUT	NPHP	*NPHP1* (NM_001128178.3)	AR				Transplant, Surveillance of extrarenal
			Deletion:Exon1-20 (HOM;p,het;m,het)		0	0	P;DM;PMID;291 46700	manifestation including dysplastic phenotype of lvier, eye and central nervous system
3010120	CAKUT	syndromic CAKUT	*PAX2* (NM_000278.5)	AD				Transplant, Ophthalmological evaluation
			c.76dup;p.Val26fs (het;p,wt;m,wt)		0	0	P;DM;PMID;858 9702	to identify and address any potential visual problems in probands and other carriers in the family
100322	CAKUT	NPHP	*NPHP1* (NM_001128178.3)	AR				Transplant, Surveillance of extrarenal
			Deletion:Exon1-20 (HOM;p,het;m,het)		0	0	P;DM;PMID;291 46700	manifestation including dysplastic phenotype of lvier, eye and central nervous system
100358	CAKUT	syndromic CAKUT	*EYA1* (NM_000503.6)	AD				Transplant Surveillance of extrarenal
			c.1545T>G;p.Tyr515Ter (het;p,wt;m,wt)		0	0	P;N	manifestation
100368	CAKUT	NPHP	*NPHP1* (NM_001128178.3)	AR				Transplant, Surveillance of extrarenal
			Deletion:Exon1-20 (HOM; p,het;m,het)		0	0	P;DM;PMID;291 46700	manifestation including dysplastic phenotype of lvier, eye and central nervous system
2065	CAKUT	syndromic CAKUT	*PAX2* (NM_000278.5)	AD				Transplant, Ophthalmological evaluation
			c.337G>T;p.Glu113Ter (het;p,wt;m,wt)		0	0	P;N	to identify and address any potential visual problems in probands and other carriers in the family
2083	Renal cystic disease	NPHP	*NPHP3* (NM_153240)	AR				Transplant, Surveillance of hepatic
			c.3757C>G;p.Leu1253Val (het;p,het;m,wt)		0.000027859	0.00016	VUS;DM;PMID;2 7491411	function impairment and liver cirrhosis post transplantation
			c.2694-2_2694-1del (het;p,wt;m,het)		0.00027457	0	LP(PVS1,PM2_p);	
100046	SRNS/FSGS	Genetic podocytopathies	*COQ8B* (NM_024876.4)	AR				Transplant, continued pharmacological
			c.1035+3A>G (het;p,het;m,wt)		0	0	VUS;N	treatment of CoQ10 supplements
			c.893+2T>A (het;p,wt;m,het)		0.00001199	0.00016	LP(PVS1, PM2_p);N	
210917	Renal cystic disease	NPHP	*NPHP1* (NM_001128178.3)	AR				Transplant, Surveillance of extrarenal
			Deletion:Exon1-20 (HOM;p,het;m,het)		0	0	P;DM;PMID;291 46700	manifestation including dysplastic phenotype of lvier, eye and central nervous system
210939	Renal cystic disease	NPHP	*NPHP1* (NM_001128178.3)	AR				Transplant, Surveillance of extrarenal
			Deletion:Exon1-20 (HOM; p,het;m,het)		0	0	P;DM;PMID;291 46700	manifestation including dysplastic phenotype of lvier, eye and central nervous system
100279	Renal cystic disease	NPHP	*NPHP1* (NM_001128178.3)	AR				Transplant, Surveillance of extrarenal
			Deletion:Exon1-20 (HOM; p,het;m,het)		0	0	P;DM;PMID;291 46700	manifestation including dysplastic phenotype of lvier, eye and central nervous system
100297	CAKUT	syndromic CAKUT	*PAX2* (NM_000278.5)	AD				Transplant, Ophthalmological evaluation
			Deletion:Exon2-3 (het;p,wt; m,wt)		0	0	P;N	to identify and address any potential visual problems in probands and other carriers in the family
100311	Renal cystic disease	NPHP	*NPHP3* (NM_153240)	AR				Transplant, Surveillance of hepatic function
			c.3775C>T;p.Arg1259Ter (het;p,het;m,wt);		0.00006	0	P;DM;PMID;180 76122	impairment and liver cirrhosis post transplantation
			c.2694-2_2694-1del (het;p,wt;m,het)		0.0004	0	P(PVS1,PM2_p, PM3);DM;PMID;	
100340	Renal cystic disease	NPHP	*NPHP1* (NM_001128178.3)	AR				Transplant, Surveillance of extrarenal
			Deletion:Exon1-20 (HOM; p,het;m,het)		0	0	P;DM;PMID;291 46700	manifestation including dysplastic phenotype of lvier, eye and central nervous system
100387	Renal cystic disease	NPHP	*NPHP1* (NM_001128178.3)	AR				Transplant, Surveillance of extrarenal
			Deletion:Exon1-20 (HOM; p,het;m,het)		0	0	P;DM;PMID;291 46700	manifestation including dysplastic phenotype of lvier, eye and central nervous system
2093	Tubulopathy	NPHP	*NPHP1* (NM_001128178.3)	AR				Transplant, Surveillance of extrarenal
			c.361_362dup;p.Ser121fs (het;p,NA;m,het)		0	0	LP;N	manifestation including dysplastic phenotype of lvier, eye and central nervous system
100341	Tubulopathy	NPHP	*NPHP4* (NM_015102.5)	AR				Transplant, Surveillance of extrarenal
			c.1462C>T;p.Arg488Ter (het;p,het;m,wt)		0.00006503	0	P;DM;PMID;157 76426	manifestation including dysplastic phenotype of lvier, eye and CNS
			c.2039C>T;p.Thr680Met (het;p,wt;m,het)		0.00004987	0	VUS(PM2_p,PM 3,PP3);N	
100012	ESRDu	Genetic podocytopathies	*COQ8B* (NM_024876.4)	AR				Transplant, continued pharmacological
			c.737G>A;p.Ser246Asn (het;p,het;m,wt);		0.00006401	0.0009	P;DM;PMID;282 04945	treatment of CoQ10 supplements
			c.1468C>T;p.Arg490Cys (het;p,wt;m,het)		0.00005843	0.00018	P(PM2_p,PM3_v erystrong,PP1,P	
100018	ESRDu	syndromic CAKUT	*PAX2* (NM_000278.5)	AD				Transplant, Ophthalmological evaluation to
			c.212+1G>T (het;p,wt;m,wt)		0	0	P;N	identify and address any potential visual problems in probands and other carriers in the family
100108	ESRDu	NPHP	*TTC21B* (NM_024753.5)	AD				Transplant, Surveillance of extrarenal
			c.553-2A>T (het;p,het;m,wt);		0	0	P;DM;PMID;333 23469	manifestation including dysplastic phenotype of lvier, eye and CNS
			c.1552T>C; p.Cys518Arg (het;p,wt;m,het)		0.000003987	0	P(PM2_p,PM3_ strong,PP1,PP3,	
2073	ESRDu	Nephrolithiasis	*SLC34A1* (NM_003052.5)	AD				Transplant, Pharmacological treatment for osteoporosis
			c.644+2T>G (het;p,wt;m,wt)		0.000203	0	P;DM;PMID;260 47794	

2100	ESRDu	syndromic CAKUT	*SALL1* (NM_002968.3)	AD				Transplant, Surveillance of hearing problem
			c.2283_2284del;p.Leu762fs (het;p,wt;m,wt)		0	0	P;N	
2104	ESRDu	Genetic podocytopathies	*COQ8B* (NM_024876.4)	AR				Transplant, continued pharmacological
			c.737G>A;p.Ser246Asn (HOM; p,het;m,het)		0.00006401	0.0009	P;DM;PMID;282 04945	treatment of CoQ10 supplements
2116	ESRDu	Genetic podocytopathies	*WT1* (NM_024426.6)	AD				Transplant, Surveillance of tumor
			c.392C>T;p.Pro131Leu (het; p,wt;m,wt)		0	0	LP;N	
210910	ESRDu	syndromic CAKUT	*PAX2* (NM_000278.5)	AD				Transplant, Ophthalmological evaluation
			c.76dup;p.Val26fs (het;p, wt; m, wt)		0	0	P;DM;PMID;858 9702	to identify and address any potential visual problems in probands and other carriers in the family
210921	ESRDu	Collagenopathies	*COL4A5* (NM_033380.3)	XLD				Transplant and Surveillance
			c.703C>T;p.Gln235Ter (hemi;p,wt;m,het)		0	0	LP;N	
210929	ESRDu	Genetic podocytopathies	*WT1* (NM_024426.6)	AD				Transplant, Surveillance of tumor
			c.1489G>C;p.Asp497His (het;p,wt;m,wt)		0	0	LP;N	
3010162	ESRDu	Collagenopathies	*COL4A4* (NM_000092.5)	AR				Transplant and Surveillance
			c.1724G>A;p.Gly575Glu (het;p,het;m,wt)		0	0	VUS;N	
			C.3636_3637del;p.Gly1213fs (het;p,wt;m,het)		0	0	LP(PVS1,PM2_p);N	
100294	ESRDu	NPHP	*NPHP1* (NM_001128178.3)	AR				Transplant, Surveillance of extrarenal
			Deletion:Exon1-20 (HOM; p,het;m,het)		0	0	P;DM;PMID;291 46700	manifestation including dysplastic phenotype of lvier, eye and central nervous system
100308	ESRDu	NPHP	*NPHP1* (NM_001128178.3)	AR				Transplant, Surveillance of extrarenal
			Deletion Exon1-20 (HOM; p,het;m,het)		0	0	P;DM;PMID;291 46700	manifestation including dysplastic phenotype of lvier, eye and central nervous system
100314	ESRDu	syndromic CAKUT	*PAX2* (NM_000278.5)	AD				Transplant, Ophthalmological evaluation
			c.213-8C>A (het;p,wt;m,wt)		0	0	LP;N	to identify and address any potential visual problems in probands and other carriers in the family
100363	ESRDu	NPHP	*ANKS6* (NM_173551.5)	AR				Transplant, Surveillance of extrarenal
			c.2394+1G>A (het;p,het;m,wt);		0	0	LP;DM;PMID;33 323469	manifestation including dysplastic phenotype of lvier and central nervous system
			c.1617G>A;p.Met539Ile (het;p,wt;m,het)		0.00001068	0	VUS(PM2_p,PM 3,PP3);N	
100377	ESRDu	Genetic podocytopathies	COQ8B(NM_024876.4)	AR				Transplant, continued pharmacological
			c.33del;p.Thr12fs (het;p,het;m,wt);		0.000058435	0.00018	P;N	treatment of CoQ10 supplements
			c.737G>A;p.Ser246Asn (het;p,wt;m,het)				P(PM2_p,PM3_v erystrong,PP1,P	
100389	ESRDu	NPHP	*NPHP1* (NM_001128178.3)	AR				Transplant, Surveillance of extrarenal
			Deletion:Exon1-20 (HOM; p,het;m,het)		0	0	P;DM;PMID;291 46700	manifestation including dysplastic phenotype of lvier, eye and central nervous system
100397	ESRDu	syndromic CAKUT	*PAX2* (NM_000278.5)	AD				Transplant, Ophthalmological evaluation
			c.70dupG;p.Leu23fs (het;p,wt;m,wt)		0	0	P;N	to identify and address any potential visual problems in probands and other carriers in the family
100393	ESRDu	Genetic podocytopathies	*COQ8B*(NM_024876.4)	AR				Transplant, continued pharmacological
			c.538C>T; p.Arg180Cys (het;p,het;m,wt);		0.000024216	0.00016	VUS;DM;PMC74 34746	treatment of CoQ10 supplements
			c.1468C>T;p.Arg490Cys (het;p,wt;m,het)		0.000058435	0.00018	P(PM2_p,PM3 _verystrong,PP1	
100353	CAKUT	syndromic CAKUT	*SALL1* (NM_002968.3)	AD				Transplant, Surveillance of hearing problem
			c.826C>T;p.Arg276Ter (het;p,wt;m,wt)		0	0	P;N	
100309	SRNS/FSGS	Genetic podocytopathies	*COQ8B* (NM_024876.4)	AR				Transplant, continued pharmacological
			c.737G>A;p.Ser246Asn (HOM; p,het;m,het)		0.00006401	0.0009	P;DM;PMID;282 04945	treatment of CoQ10 supplements
100293	SRNS/FSGS	Genetic podocytopathies	*NPHS1* (NM_004646.4)	AR				Transplant and Surveillance
			c.2512G>T; p.Pro838Thr (het; p, het; m, wt)		0	0	VUS ; DM; 28780565	
			c.803C>T; p. Arg268Gln (het; p, wt; m, het)		0.00129	0.01283	VUS (PM2_p,PP3);N	
100328	SRNS/FSGS	Genetic podocytopathies	*WT1* (NM_024426.6)	AD				Transplant and Surveillance
			c.1244A>C;p.His415Pro (het; p,NA;m,NA)		0	0	VUS;DM;CM002 419	
100345	SRNS/FSGS	Genetic podocytopathies	*NUP107* (NM_020401.4)	AR				Transplant and Surveillance
			c.1450_1452delCAT;p.Gly484del (het; p, het; m, wt)		0	0	VUS;N	
			c.727T>G; p. Trp243Gly (het; p, wt; m, het)		0	0	VUS(PM2_p,PP 3);N	
100023	Nephritis	NPHP	*TTC21B*(NM_024753.5)	AR				Transplant and Surveillance
			c.1552T>C:p.Cys518Arg;(het; p,het;m,wt)		0	0	P(PM2_p,PM3 _strong,PP1,PP	
			c.1A>G:p.Met1Val(het; p,wt;m,het)		0.00001357	9.20E-05	VUS(PM2_p,PM 3,PP3);N	

gnomAD, variant frequencies listed for homozygous/ hemizygous (if applicable)/ heterozygous/ total alleles(http://gnomad.broadinstitute.org/). All, all population, EA, eastern Asian.

HGMD, Human Gene Mutation Database(https://portal.biobaseinternational.com/hgmd). If the exact variant has been reported previously on HGMD® Professional 2017. For the reported phenotype and classified as a disease-
causing pathogenic mutation, the variant is denoted as “DM.” The variant is denoted as “LD” if the variant is likely a disease-causing pathogenic mutation, but either the author indicated some doubt or subsequent evidence calls the
deleterious nature of the variant into question. If the gene, but not the exact variant, has been reported for the corresponding phenotype, then “N” is indicated in this column.
ACMG, American College of Medical Genetics and
Genomics Standards and Guidelines Classification as pathogenic, likely pathogenic or VUS (Richards Genet Med 17(5):405, 2015).

NPHP: nephronophthisis; FSGS:focal segmental glomerulosclerosis; PKD: polycystic kidney disease; CAKUT: congenital abnormalities of the kidney and urinary tract; SRNS: steroid-resistant nephrotic syndrome; KFu: kidney failure of unknown etiology

Table S3: Variants of uncertain significance and secondary findings

Family ID	A Priori Clinical Diagnosis	Inherited pattern	Gene; c. Change[a]; p. Change[b]; Segregation (p, m)	gnomAD (All)[c]	gnomAD (EA)[d]	ACMG (HGMD)
			NPHS1 (NM_004646.4)			
100293	SRNS	AR	c.2512G>T; p.Pro838Thr; (het; p, het; m, wt);	0	0	VUS (PM2_p,PP3);DM;28780565
			c.803C>T; p. Arg268Gln; (het; p, wt; m, het)	0	0	VUS (PM2_p,PP3);N
100328	FSGS	AD	*WT1* (NM_024426.6)			
			c.1244A>C;p.His415Pro; het; (p,NA;m,NA)	0	0	VUS(PM2_p,PP3);DM;CM002419
			NUP107 (NM_020401.4)			
100345	SRNS	AR	c.1450-1452delCAT; (p, het; m, wt);	0	0	VUS(PM2_p);N
			c.727T>G; p. Trp243Gly; (het; p, wt; m, het)	0	0	VUS(PM2_p,PP3);N
			NUP93 (NM_014669.5)			
100362	SRNS	AR	c.1573C>T; p. Arg525Trp; (het; p, het; m, wt);	0	0	VUS (PM2_p,PP3); DM; CM1916292
			c.1899G>A; p. Lys633=; (het; p, wt; m, het)	0	0	VUS(PM2_p);N
			PKHD1 (NM_138694.3)			
100378	PKD	AR	c.5918T>C; p. Leu1973Pro; (het; p, het; m, wt);	0	0	DM;VUS (PM2_p, PP3);
			c.7994T>C; p. Leu2665Pro; (het; p, wt; m, het)	0	0	VUS(PM2_p)
			WDR1 (NM_017491.5)			
100380	PKD	AR	c.1993G>C; p. Ala665Pro; (p, het; m, wt);	0	0	VUS(PM2_p);N
			c.2489G>A; p. Gly830Glu; (het; p, wt; m, het)	0	0	VUS(PM2_p, PP3);N
100367	SRNS	AR	*CRB2* (NM_173689.7)			
			c.1813C>T;p.Arg605Cys;(p,het;m,het)	0	0	VUS(PM1,PM2_p,PP3);N
			NPHP3 (NM_153240)			
2075	PKD	AR	c.425C>G; p. Thr142Ser; (het; p, NA; m, NA);	7.98E-06	5.44E-05	VUS(PM2_p,PP3);N
			c.1942G>A; p. Val648Ile; (het; p, NA; m, NA)	0.000395	0.003238	VUS (PM2_p);N
2098	FSGS	AD	*PKD1* (NM_001009944.3)			
			c.1585G>A; p. Val529Ile; (het; p, NA; m, NA)	5.9E-05	0.000372	DM; VUS (PM2_p)
2102	ESRDu	AD	*PKD1* (NM_001009944.3)			
			c.4789A>G; p. Ile1597Val; (het; p, NA; m, NA)	8.02E-06	0.000109	DM; VUS (PM2_p)
			PAX2(NM_000278.5)			
210907	ESRDu	AD				

210907	ESRDu	AD	c.154T>C; p. Cys52Arg; (het; p, wt; m, het)	0	0	VUS(PM2_p);N
210911	Nephritis	AD	*INF2* (NM_022489.4)			
			c.383T>C; p. Leu128Pro; (het; p, NA; m, NA)	0	0	DM; VUS (PM2_p,PP3)
210927	Nephritis	XLD	*COL4A5* (NM_033380.3)			
			c.1303G>C; p. Gly435Arg; (het; p,wt; m,het)	0	0	VUS(PM2_p,PP3);N
			TMEM67 (NM_153704.6)			
2010103	ESRDu	AR	c.224-3delT; (het; p, NA; m, NA);	0	0	DM; VUS(PM2_p,PP3);
			c.725A>G; p. Asn242Ser; (het; p, NA; m, NA)	0	0	VUS(PM2_p)
			TTC21B (NM_024753.5)			
3010117	ESRDu	AR	c.380C>T; p.Ala127Val;(het; p, het; m, wt);	0	0	VUS(PM2_p,PP3);
			c.2211+3A>G;(het; p, wt; m, het)	0	0	VUS(PM2_p,PP3)
210915	CAKUT	AD	*WNK1* (NM_213655.5)			
			c.1748dup;p.Gln584fs;(het;p,NA;m,het)	0.000452	8.87E-05	VUS(PVS1);N
100337	ESRDu		*WT1* (NM_024426.6)			
			c.745+4C>T;(het;p,wt;m,wt)	0	0	VUS(PM2_p);N
210907	ESRDu	AD	*PAX2* (NM_000278.5)			
			c.154T>C; p.Cys52Arg (het; p, wt; m, het)	0	0	VUS;N
2103	Nephritis	AR	*ANKS6* (NM_173551.5)			
			c.887T>C;p.Ile296Thr (het;p,het;m,wt)	0	0	VUS;DM; PMID;30180410
			c.651dupC;p.Asn218fs (het;p,wt;m,het)	0	0	P(PVS1,PM2_p,PM3);DM;PMID;30180410
100307	SRNS/FSGS	AD	*CFHR1* (NM_002113.3)			
			c.208A>G;p.Ile70Val (HOM; p,het,m,wt)	0	0	LB (PM2, BS2, BP4);N
			Deletion:Exon1-6 (het; p,wt;m,het)			LP(PVS1,PM2_p);N
100369	CAKUT	AD	*WT1* (NM_024426.6)			
			11p14.1p12 del (het; p,wt;m,wt)	0	0	P;N
100355	CAKUT	XLR	*COL4A5* (NM_033380.3)			
			c.1207G>A;p.Gly403Arg (Hemi;p,wt;m,het)	0	0	LP;N
100407	CAKUT	XLR	*COL4A5* (NM_033380.3)			
			c.1226G>A;p.Gly409Asp (Hemi;p,NA;m,NA)	0	0	P;DM;PMID;8651296
2094	CAKUT	XLR	*WT1* (NM_024426.6)			
			c.1432+4C>T (het;p,wt;m,wt)	0	0	LP;N

3010168	Renal cystic disease	AR	*COL4A4* (NM_000092.5)			
			c.1724G>A;p.Gly575Glu (het; p,het;m,wt)	0	0	VUS;N
			c.3636_3637del;p.Gly1213fs (het; p,wt;m,het)	0	0	LP(PVS1,PM2_p);N
100290	Renal cystic disease	AR	*MMACHC* (NM_015506.3)			
			c.683C>T;p.Ala228Val (het;p,het;m,wt)	0.0024	0.0006	VUS;DM;PMID;30157807
			c.609G>A;p.Trp203Ter (het;p,wt;m,het)	0	0	P(PVS1,PM2_p,PM3);DM;PMID;16311595
2090	CAKUT	AD	*TNXB* (NM_001365276.2)			
			c.12469+2T>C (het;p,wt;m,wt)	0.01142	0.00092	P;N
3010166	Tubulopathy	AR	*CTNS* (NM_004937.3)			
			c.1054C>T;p.Gln352Ter (het;p,het;m,wt)	0	0	P;N
			c.314_317del;p.His105fs (het;p,wt;m,het)	0	0	P(PVS1,PM2_p,PM3, PP4);DM;PMID;9792862
210903	Renal cystic disease	AR	*CFTR* (NM_000492.4)			
			c.2158C>T;p.Gln720Ter (het;p,NA;m,NA)	3.98E-06	0	LP;N
			c.2909G>A;p.Gly970Asp (het;p,NA;m,NA)	1.2E-05	0.00016	LP(PM2_p,PM3 _strong,PP3); DM;PMID;10453741
2075	Renal cystic disease	AR	*NPHP3* (NM_153240)			
			c.425C>G; p.Thr142Ser (het; p, NA; m, NA)	7.98E-06	5.40E-05	VUS;N
			c.1942G>A; p.Val648Ile (het; p, NA; m, NA)	0.000395	0.00324	VUS (PM2_p);N
2102	ESRDu	AD	*PKD1* (NM_001009944.3)			
			c.4789A>G; p. Ile1597Val (het; p, NA; m, NA)	8.02E-06	0.00011	DM; VUS

a Impact of variant on cDNA level.; Impact of variant on the amino acid or protein level.
b gnomAD, variant frequencies listed for homozygous/ hemizygous (if applicable)/ heterozygous/ total alleles(http://gnomad.broadinstitute.org/). All, all population, EA, eastern Asian.
c HGMD, Human Gene Mutation Database(https://portal.biobaseinternational.com/hgmd). If the exact variant has been reported previously on HGMD® Professional 2017.2 for the reported phenotype and classified as
